# Data-driven, cross-disciplinary collaboration: lessons learned at the largest academic health center in Latin America during the COVID-19 pandemic

**DOI:** 10.3389/fpubh.2024.1369129

**Published:** 2024-02-27

**Authors:** Ana Paula Ritto, Adriana Ladeira de Araujo, Carlos Roberto Ribeiro de Carvalho, Heraldo Possolo De Souza, Patricia Manga e Silva Favaretto, Vivian Renata Boldrim Saboya, Michelle Louvaes Garcia, Leslie Domenici Kulikowski, Esper Georges Kallás, Antonio José Rodrigues Pereira, Vilson Cobello Junior, Katia Regina Silva, Eidi Raquel Franco Abdalla, Aluisio Augusto Cotrim Segurado, Ester Cerdeira Sabino, Ulysses Ribeiro Junior, Rossana Pulcineli Vieira Francisco, Anna Miethke-Morais, Anna Sara Shafferman Levin, Marcio Valente Yamada Sawamura, Juliana Carvalho Ferreira, Clovis Artur Silva, Thais Mauad, Nelson da Cruz Gouveia, Leila Suemi Harima Letaif, Marco Antonio Bego, Linamara Rizzo Battistella, Alberto José da Silva Duarte, Marilia Cerqueira Leite Seelaender, Julio Marchini, Orestes Vicente Forlenza, Vanderson Geraldo Rocha, Maria Cassia Mendes-Correa, Silvia Figueiredo Costa, Giovanni Guido Cerri, Eloisa Silva Dutra de Oliveira Bonfá, Roger Chammas, Tarcisio Eloy Pessoa de Barros Filho, Geraldo Busatto Filho

**Affiliations:** ^1^Faculdade de Medicina, Hospital das Clínicas HC-FMUSP, Universidade de São Paulo, São Paulo, Brazil; ^2^Faculdade de Medicina, Instituto do Coração, Hospital das Clínicas HC-FMUSP, Universidade de São Paulo, São Paulo, Brazil; ^3^Departamento de Emergências Médicas, Faculdade de Medicina, Hospital das Clínicas HC-FMUSP, Universidade de São Paulo, São Paulo, Brazil; ^4^Diretoria Executiva dos Laboratórios de Investigação Médica, Faculdade de Medicina da Universidade de São Paulo, São Paulo, Brazil; ^5^Departamento de Patologia, Faculdade de Medicina da Universidade de São Paulo, São Paulo, Brazil; ^6^Departamento de Moléstias Infecciosas e Parasitárias, Hospital das Clínicas HC-FMUSP, Faculdade de Medicina da Universidade de São Paulo, São Paulo, Brazil; ^7^Núcleo Especializado em Tecnologia da Informação, Hospital das Clínicas HC-FMUSP, Faculdade de Medicina da Universidade de São Paulo, São Paulo, Brazil; ^8^Divisão de Biblioteca e Documentação, Faculdade de Medicina da Universidade de São Paulo, São Paulo, Brazil; ^9^Departamento de Gastroenterologia, Hospital das Clínicas HC-FMUSP, Faculdade de Medicina da Universidade de São Paulo, São Paulo, Brazil; ^10^Departamento de Obstetrícia e Ginecologia, Hospital das Clínicas HC-FMUSP, Faculdade de Medicina da Universidade de São Paulo, São Paulo, Brazil; ^11^Diretoria Clínica, Hospital das Clínicas HC-FMUSP, Faculdade de Medicina da Universidade de São Paulo, São Paulo, Brazil; ^12^Faculdade de Medicina, Instituto de Radiologia, Hospital das Clínicas HC-FMUSP, Universidade de São Paulo, São Paulo, SP, Brazil; ^13^Instituto da Criança e do Adolescente, Hospital das Clínicas HC-FMUSP, Faculdade de Medicina da Universidade de São Paulo, São Paulo, Brazil; ^14^Departamento de Medicina Preventiva, Faculdade de Medicina da Universidade de São Paulo, São Paulo, Brazil; ^15^Instituto de Medicina Física e Reabilitação, Hospital das Clínicas HC-FMUSP, Faculdade de Medicina da Universidade de São Paulo, São Paulo, Brazil; ^16^Divisão de Laboratório Central, Hospital das Clínicas HC-FMUSP, Faculdade de Medicina da Universidade de São Paulo, São Paulo, Brazil; ^17^Departamento de Cirurgia, Hospital das Clínicas HC-FMUSP, Faculdade de Medicina da Universidade de São Paulo, São Paulo, Brazil; ^18^Departamento e Instituto de Psiquiatria, Hospital das Clínicas HC-FMUSP, Faculdade de Medicina, Universidade de São Paulo, São Paulo, Brazil; ^19^Departamento de Clínica Médica, Hospital das Clínicas HC-FMUSP, Faculdade de Medicina, Universidade de São Paulo, São Paulo, Brazil; ^20^Departamento de Radiologia e Oncologia, Faculdade de Medicina, Universidade de São Paulo, São Paulo, Brazil; ^21^Departamento de Ortopedia e Traumatologia, Hospital das Clínicas HC-FMUSP, Faculdade de Medicina, Universidade de São Paulo, São Paulo, Brazil

**Keywords:** COVID-19, cross-disciplinarity, research collaboration, research management, research data, data science, data management, health data analysis

## Abstract

**Introduction:**

The COVID-19 pandemic has prompted global research efforts to reduce infection impact, highlighting the potential of cross-disciplinary collaboration to enhance research quality and efficiency.

**Methods:**

At the FMUSP-HC academic health system, we implemented innovative flow management routines for collecting, organizing and analyzing demographic data, COVID-related data and biological materials from over 4,500 patients with confirmed SARS-CoV-2 infection hospitalized from 2020 to 2022. This strategy was mainly planned in three areas: organizing a database with data from the hospitalizations; setting-up a multidisciplinary taskforce to conduct follow-up assessments after discharge; and organizing a biobank. Additionally, a COVID-19 curated collection was created within the institutional digital library of academic papers to map the research output.

**Results:**

Over the course of the experience, the possible benefits and challenges of this type of research support approach were identified and discussed, leading to a set of recommended strategies to enhance collaboration within the research institution. Demographic and clinical data from COVID-19 hospitalizations were compiled in a database including adults and a minority of children and adolescents with laboratory confirmed COVID-19, covering 2020–2022, with approximately 350 fields per patient. To date, this database has been used in 16 published studies. Additionally, we assessed 700 adults 6 to 11 months after hospitalization through comprehensive, multidisciplinary in-person evaluations; this database, comprising around 2000 fields per subject, was used in 15 publications. Furthermore, thousands of blood samples collected during the acute phase and follow-up assessments remain stored for future investigations. To date, more than 3,700 aliquots have been used in ongoing research investigating various aspects of COVID-19. Lastly, the mapping of the overall research output revealed that between 2020 and 2022 our academic system produced 1,394 scientific articles on COVID-19.

**Discussion:**

Research is a crucial component of an effective epidemic response, and the preparation process should include a well-defined plan for organizing and sharing resources. The initiatives described in the present paper were successful in our aim to foster large-scale research in our institution. Although a single model may not be appropriate for all contexts, cross-disciplinary collaboration and open data sharing should make health research systems more efficient to generate the best evidence.

## Introduction

The COVID-19 pandemic, caused by the SARS-CoV-2 infection, has triggered an urgent global research effort to mitigate its impact. At the time, it was essential to devise effective strategies to reduce the rate, severity, and economic aftermath of SARS-CoV-2 infection (1–3). This scenario persists, owing to the recognition that many patients present a post-acute COVID-19 syndrome (PACS), which can appear as an intricate combination of persisting and novel symptoms (2). In recent years, collaboration among healthcare researchers with diverse expertise and access to large-scale patient data has emerged as a critical approach to enhancing research quality and efficiency (4–11). Cross-disciplinary collaboration involves a joint and equal contribution from a broad range of health research experts, crossing disciplinary boundaries to work collaboratively (12–16). This emerging view of science can and should also be employed for PACS, following the efforts during the acute phase of the pandemic.

In response to the COVID-19 outbreak in Brazil (one of the most affected countries), the *Hospital das Clínicas & Faculdade de Medicina da Universidade de São Paulo* (HC-FMUSP), the largest academic health system in Latin America, established a crisis committee in January 2020. Over the following 2 years, the HC-FMUSP complex admitted over 9,000 patients with suspected SARS-CoV-2 infection, mostly moderate and severe cases. To cope with surge in demand for COVID-19 care during the first wave of the pandemic (from March through August 2020), the crisis committee converted the Central Institute, one of the eight HC-FMUSP institutes, into a specialized COVID-19 inpatient facility, with a total of 900 beds (including 300 intensive care beds) (17, 18).

At the onset of the pandemic, several research groups initiated clinical studies on COVID-19 and explored various preventive strategies for the disease (19). In May 2020, HC-FMUSP installed an emergency institutional taskforce, aimed to support research infrastructure and logistics for those studies, which had until then been conducted with a low degree of connection and collaboration among teams. A set of institutional cross-disciplinary research initiatives to study and provide solutions for COVID-19 was thus implemented by this taskforce, with the purpose of fostering scientific collaborations among groups affiliated with HC-FMUSP. This enterprise was designed to reach far beyond co-authorship and, instead, involved joint institutional efforts across disciplines with a focus on cooperation, equity, and transparency (20–23). This paper aims to describe, in detail, the successful implementation of such initiatives (including flow management routines to capture, organize, share and analyze large amounts of data), and outline the challenges and barriers identified over the course of this unprecedented experience in the country.

## Materials and methods

### Context

This paper examines the benefits and challenges of an institutional research management initiative implemented to facilitate large-scale, cross-disciplinary scientific collaborations during the COVID-19 pandemic. In response to the urgent need for knowledge about the disease and the resource constraints faced during the pandemic, all the actions described below were designed and implemented simultaneously, rendering this a particularly challenging and complex endeavor.

### Overall strategy and governance

Research managing strategies were planned in three main areas: organization of a large database consisting of clinical data from hospitalized COVID-19 patients; setting-up of a multidisciplinary taskforce to conduct follow-up assessments of these patients; and organization of a biobank of blood samples collected both during inpatient stay and follow-up assessments.

A COVID-19 Steering Committee was established, comprising institutional leaders with expertise in scientific management and representatives from the COVID-19 crisis committee. This committee shared several key responsibilities, including mapping, monitoring, and supporting research groups utilizing data from the institutional databases. The committee also proposed strategies to encourage collaborative publications and approved applications from HC-FMUSP researchers seeking access to the databases. To ensure fair decision-making, the vice-chair of FMUSP has served as an adjunct member of the Steering Committee, responsible for reviewing and adjudicating appeals from dissatisfied applicants regarding committee decisions.

**Figure 1:**
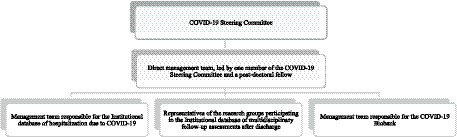
Flow-chart of HC-FMUSP research-managing structure to foster large-scale cross-disciplinary collaborative research studies on COVID-19.

To ensure effective management of the COVID-19 data organization initiatives, specific teams were created for each of the three institutional fronts. Additionally, a small team was responsible for the overall day-to-day management of these fronts. This group, led by a university full professor who was also a member of the Steering Committee, facilitated communication and collaboration, acting as a catalyst for the exchange of relevant information and intelligence related to COVID-19 research within the institution (Figure 1). With support from the FMUSP Library, this direct management team prepared an institutional data management plan for the various initiatives, which was validated by the COVID-19 Steering Committee and approved by the HC-FMUSP board of directors. This document outlined the criteria for granting access to institutional data and biological material, as well as periods of retention prior to open data sharing.

Regarding the initial financing for the research initiatives outlined in this report, the HC-FMUSP superintendence rapidly provided seed funds generated from a crowdfunding campaign launched during the pandemic^1^ (see details of funding allocation in Supplementary materials).

### Implementation of actions and collaborative data collection

#### Institutional database of hospitalizations due to COVID-19

The dedicated inpatient facility for COVID-19 patients was operational at the Central Institute of HC-FMUSP until September 2020, coinciding with the abatement of the first wave of COVID-19 cases in São Paulo. From then onwards, inpatient admissions due to acute COVID-19 during the subsequent waves of the pandemic continued to take at HC-FMUSP, being allocated to different units of the hospital complex.

**Figure 2:**
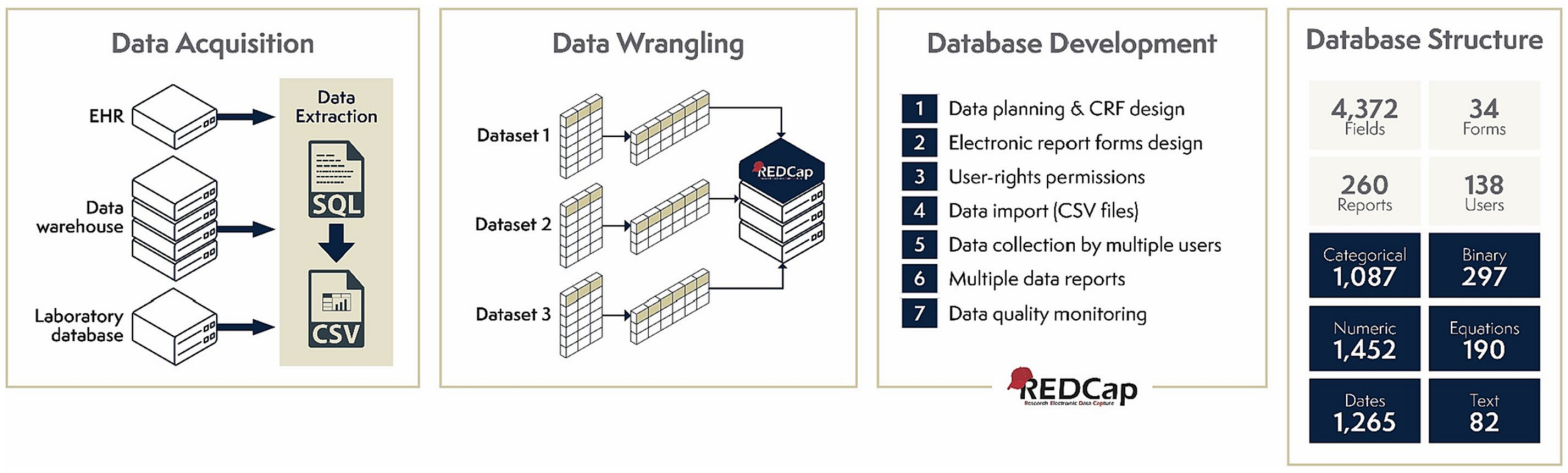
Data curation processes involved in the development of HC-FMUSP COVID-19 institutional databases. EHR, electronic health record; SQL, Structured Query Language; CSV, Comma-separated values; CRF, case report form.

In May 2020, HC-FMUSP initiated the development of the institutional database focused on hospitalization data. This database included information from consecutive patients admitted for at least 24 h as inpatients due to suspected SARS-CoV-2 infection. The HC-FMUSP Information Technology (IT) Center extracted data from structured fields within electronic health records (EHR) and populated the database. These records followed a specific case report form designed for COVID-19 within the HC-FMUSP EHR system, facilitating the collection of pertinent information during hospital admissions. The basic set of variables was defined by a panel of experts in clinical emergencies, intensive care and infectious diseases, combined with the case report form proposed by the World Health Organization to globally standardize COVID-19 records (24). Data regarding vital signs, laboratory and radiology tests, and drug prescriptions were also extracted by the IT Center, assisted by physicians to determine where the most accurate clinical information was available within the EHR. A team of data science specialists was hired to organize all data into a set of variables usable for research, further data mine EHR, and organize all the processes involved in the construction of the institutional databases (including, cleaning, structuring, and reconciliation; Figure 2). The database was stored on a Research Electronic Data Capture (REDCap) system (25) hosted at HC-FMUSP servers.

This database was further expanded with two additional sources: 1. contributions from research groups who agreed to share the data that they had already been collecting for their own studies with inpatients; and 2. information manually extracted from unstructured fields of the EHR by a taskforce of young researchers supervised by experienced scientists, in order to fill missing data for selected variables.

Gradually, this hospitalization database was integrated with information from two other institutional initiatives which are described below (i.e., the multidisciplinary follow-up assessment of COVID-19 patients after in-hospital discharge, and the COVID-19 biobank).

Direct access to data from the hospitalization database was provided solely by the direct management team, after swift authorization of the COVID-19 Steering Committee using objective and previously advertised criteria. This ensured objectivity and transparency in the process of granting access to the data.

#### Multidisciplinary follow-up assessments after discharge

The multidisciplinary follow-up assessment program of COVID-19 patients was carried out from October 2020 to April 2021, as detailed elsewhere (26). All surviving adult (≥18 years) patients that had been admitted to HC-FMUSP between March and August 2020 due to COVID-19 were consecutively invited for a follow-up visit that should occur around 6 months after their hospitalization. Comorbid conditions prior to COVID-19 were identified using the hospitalization database described in the previous sub-section, and patients with a previous diagnosis of dementia or end-stage cancer were excluded. Additional exclusion criteria were pregnant or postpartum patients, subjects living in nursing homes or long-term care facilities, and insufficient physical mobility to leave home.

The plans for this follow-up initiative were extensively advertised throughout HC-FMUSP, and all interested research groups were invited to participate. To ensure cooperation and minimize inconvenience for the patients, groups were required to collect data in an integrated and coordinated manner.

To optimize participants’ time during the in-person visit, participants of the follow-up cohort were initially evaluated remotely. All interviews and protocols that could be administered remotely were answered during this telehealth consultation, taking advantage of the infrastructure and training of health care professionals that were implemented for innovative tele-ICU practices during the pandemic at HC-FMUSP (27–29). Most in-person assessments were streamlined on a single day, approximately 1 week after the remote evaluation, optimizing the use of institutional resources, maximizing multidisciplinary interchange of experiences, and fostering a comprehensive outlook on the health needs of the subjects who underwent those follow-up assessments. Participants with a history of ICU admission and diagnosed with lung damage during hospitalization, according to predefined criteria (30, 31), were invited for a second visit to undergo specific tests (plethysmography, cardiac stress test and chest computed tomography). To facilitate interactions between research groups and to avoid the physical circulation of subjects and their relatives, all in-person evaluations (except radiological exams) were conducted at one single hospital sector. Two separate facilities were used: a temporary outpatient center for patients without a history of ICU admission during in-hospital stay and the clinical research center at the *Instituto do Coração* at HC-FMUSP for patients who had been admitted to an ICU during acute COVID-19 (26).

The multidisciplinary follow-up assessment also included the evaluation of hospitalized pediatric COVID-19 patients (<18 years), specifically focusing on multisystem inflammatory syndrome in children (MIS-C) (32). To facilitate this assessment, a dedicated outpatient clinic was established at HC-FMUSP’s Children’s Institute, where patients were scheduled for visits every 6 months. The prospective studies conducted on children and adolescents that had COVID-19 encompassed various areas, such as linear and pubertal development, dietary habits, mental health, innate immunity errors, autoimmune conditions, metabolomics, gut microbiota, genetic determinants, bone mineral density, and home-based exercise training (33).

Collected data were stored on the REDCap system hosted at HC-FMUSP, fully integrated with the hospitalization database. Access to those data was provided solely but with swift authorization by the direct management team. The variables that could be accessed by each participating team and the principles for the collaborative sharing of information were agreed between those groups. Information on periods of retention for the broader sharing of those data was included in the institutional data management plan.

#### COVID-19 biobank

Our institution’s COVID-related activities included a pioneering effort to collect and store large amounts of biological material from hospitalized COVID-19 patients for both short-term and future scientific studies. This initiative utilized an existing biobank at the Tropical Medicine Institute of HC-FMUSP, which had prior approval from the Brazilian Council of Ethics in Research to incorporate residual biological material from diagnostic samples collected during routine clinical procedures at HC-FMUSP, with explicit patient consent. A dedicated COVID-19 branch of the biobank was established at the Central Laboratory of HC-FMUSP, allowing for the systematic processing and storage of leftover blood samples collected from hospitalized COVID-19 patients starting in June 2020.

#### Creation of a COVID-19 curated collection within the institutional digital library of academic papers at the FMUSP-HC system

Using DSpace software and in line with the institution’s strategic needs, the FMUSP-HC Library developed a COVID-19 curated collection within the Intellectual Production Observatory of the FMUSP-HC academic system – OPI.^2^ OPI is an institutional digital library of academic papers created in 2014 to facilitate the mapping, monitoring and analyzing of quantitative metrics related to the research output of FMUSP-HC groups.

##### Ethical approval, consent and data security aspects

The implementation of all actions described in this paper strictly followed ethical and data security principles, adhering to standards of consent, privacy, confidentiality, and data protection. All research protocols included in the initiatives described herein received ethical approval. The multidisciplinary follow-up cohort integrates the results of several research projects led by health specialist teams within HC-FMUSP. All projects were approved by HC-FMUSP’s institutional review board (CAPPesq – *Comissão de Ética para Análise de Projetos de Pesquisa*) (approval numbers 4.270.242, 4.502.334, 4.524.031, 4.302.745 and 4.391.560). Participants provided signed informed consent.

In 2020, voluntary medical students made efforts to obtain informed consent for the COVID-19 biobank from hospitalized individuals and their relatives during their inpatient stay. These efforts were continued through subsequent telephone and face-to-face contacts during the follow-up program.

To ensure data security and confidentiality, the REDCap system hosted at HC-FMUSP complies with U.S. Health Insurance Portability and Accountability Act (HIPAA) and the Brazilian General Personal Data Protection Act (in Portuguese, LGPD). Researchers accessing data and samples are required to sign agreements acknowledging the ethical and legal responsibilities and ensuring strict confidentiality of participants’ data.

## Results

### Institutional databases

#### Institutional database of hospitalizations due to COVID-19

Data from COVID-19 hospitalizations were consistently extracted and compiled in the research database from all disease waves, through June 2022. By that date, the institutional research database contained hospitalization data from more than 4,500 adults with laboratory-confirmed diagnosis of COVID-19, including cases from March 2020 to June 2022 (see Table 1; Supplementary Table S1), with approximately 350 fields from each patient (see Supplementary Table S2). The pediatric database including hospitalization data from more than 150 children and adolescents admitted to HC-FMUSP due to COVID-19 was organized by research groups based at the specialized HC-FMUSP’s Children’s Institute.

**Table 1:** Baseline and hospitalization characteristics of adult patients (≥ 18 years) with confirmed SARS-Cov-2 infection hospitalized from 2020 to 2022 available in our database.

Hospitalization database	
Laboratory-confirmed COVID-19 cases^a^	N = 4,686
Age – mean (±standard deviation)	58.5 (±16.2)
Sex – *N* (%)	
Female	2,140 (45.7%)
Male	2,546 (54.3%)
	
Charlson comorbidity score – mean (±standard deviation)	3.3 (±2.1)
WHO clinical progression scale^b^ – frequency in different categories	
3–4	671 (14.3%)
5	1,605 (34.2%)
6	181 (3.9%)
7–8-9	2,229 (47.6%)
Events during hospitalization	
Hospital stay, duration in days – mean (±standard deviation)	16.2 (±15.9)
Admission to intensive care unit (ICU) – *N* (%)	3,227 (68.9%)
Intubation – *N* (%)	2,230 (47.6%)
Renal replacement therapy – *N* (%)	956 (20.4%)
In-hospital death – *N* (%)	1,501 (32.0%)

^a^Either: (1) positive reverse-transcriptase polymerase chain reaction (RT-PCR) for SARS-CoV-2 on swab from nasopharyngeal and/or oropharyngeal samples (collected at admission and repeated after 48 h if negative); or (2) positive testing by chemiluminescent immunoassays to detect serum antibodies, performed for highly suspect cases with at least two negative RT-PCR samples or for whom an RT-PCR test was not available up to day 10 of symptom onset. Patients with nosocomial COVID-19 infections were excluded. ^b^WHO scale categories: 3–4, no continuous supplemental oxygen needed; 5, continuous supplemental oxygen only; 6, continuous positive airway pressure ventilation, bi-level positive airway pressure or high flow nasal oxygen; 7–8-9, invasive mechanical ventilation and/or extra-corporeal membrane oxygenation (ECMO). WHO Working Group on the Clinical Characterization and Management of COVID-19 infection (2020).

The collaborative efforts of several teams at HC-FMUSP were crucial to maximize the quality of the data compiled in the hospitalization database above. The teams from the HC-FMUSP Infectious Diseases section and the HC-FMUSP Central Laboratory were responsible for developing and overseeing the application of the criteria for laboratory-based diagnosis of COVID-19 (34, 35). The Pulmonology and Radiology teams worked to validate and apply the criteria for radiological diagnosis of COVID-19 mainly based on lung computed tomography (CT) findings. The Infectious Diseases team, together with the Pulmonology and Radiology groups, devised the clinical criteria for highly suspect cases of COVID-19 (36). Using the defined criteria, the specialized Epidemiological Surveillance team at HC-FMUSP validated the inclusion of cases in the institutional research database while excluding patients with nosocomial COVID-19 infections.

Finally, the expertise of two HC-FMUSP groups involved in environmental research allowed the generation of neighborhood variables based on each patient’s zip code of residence. These variables included factors such as air pollution levels and exposure to green areas, which were incorporated into the research database to help explore potential environmental risk factors associated with post-COVID-19 syndrome (37).

Upon completion of the database and case validation, the possibility of accessing the institutional database above was widely advertised in successive calls open to HC-FMUSP-based research groups. Thus far, the database has been used in 17 published studies, attracting several research groups (36, 38–53) (see Supplementary Table S3). The hospitalization database is currently being used for a few additional analyses, and it will continue to be accessible for new studies proposed in the near future. This unique database also provides the means for assessing long-term outcome of patients, as it provides a profusion of baseline data on the different clinical parameters, allowing the continuous horizontal follow-up of patients.

#### Multidisciplinary follow-up assessments of COVID-19 patients after hospital discharge

More than 20 HC-FMUSP-based research groups from different disciplines agreed to join the multidisciplinary follow-up assessment program, bringing human and operational resources to make the collection of comprehensive data from hundreds of patients feasible over a few months without the need for large external financial resources.

**Figure 3:**
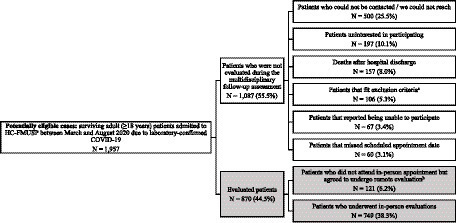
Flowchart of the multidisciplinary follow-up assessment program cohort. ^a^Exclusion criteria: previous diagnosis of dementia or end-stage cancer, pregnant or postpartum patients, patients living in nursing homes or long-term care facilities or insufficient physical mobility to leave home. ^b^Participants who consented with remote assessments but declined the invitation to attend in-person appointments due to health concerns. Despite the implementation of measures to mitigate exposure risk during follow-up evaluations, there was still a level of heightened health apprehension, since the multidisciplinary follow-up assessments occurred between October 2020 and January 2021, when the pandemic was still at its peak in São Paulo.

From October 2020 to April 2021, over 700 adults (mean age 54.8 ± 14.1 years, 53% male) were reassessed between 6 and 11 months after hospitalization due to COVID-19, using the structured multidisciplinary protocol (26) (see flowchart in Figure 3). The resulting database, comprising approximately 2000 fields for each subject (see Supplementary Table S2), has thus far been used in 15 publications (30, 31, 33, 37, 54–64) (see Supplementary Table S4), and it is continuously accessible for new studies.

This collaborative experience paved the way for an ambitious multidisciplinary grant proposal submitted to the state-run São Paulo Research Foundation (FAPESP), to fund two additional waves of follow-up assessments of the same cohort (after three and after 4 years of hospital admission), combining research proposals from the different groups involved thus far. This proposal, worth approximately 1.8 million dollars, was awarded by FAPESP at the beginning of 2023 acknowledging the potential impact of the proposed research on advancing the understanding, prevention, and treatment of COVID-19.

#### COVID-19 biobank

By October 2020, blood serum and plasma samples had been collected, processed, and stored at -80^o^ C from over 2,000 patients hospitalized due to COVID-19 at HC-FMUSP. Additional vials of serum were stored for more than 700 patients who agreed to attend the follow-up visit. In total, the biobank contains over 45,000 blood vials (each of containing approximately 0.5 mL). To date, over 3,700 aliquots have been dispensed for 10 different ongoing research initiatives investigating pathophysiological aspects of COVID-19, relating distinct acute symptoms and sequelae of the disease to a wide range of biomarkers of inflammation, neurodegeneration, intestinal permeability, peptidomics and metabolomics, among others (see Supplementary Table S5).

There was considerable variability in how these biomarker studies were conceived, including one top-down initiative led by the COVID-19 Steering Committee related to the assessment of a large panel of inflammatory markers, whereby all research groups with expertise and interest in the field at HC-FMUSP were contacted and encouraged to work collaboratively, planning and conducting analyses on hundreds of samples and sharing costs of consumables.

Up until now, two collaborative papers from the above studies have been published (65, 66).

#### COVID-19 curated collection within the institutional digital library of academic papers

By the end of 2022, the FMUSP-HC System’s researchers had authored 1,394 papers on COVID-19 published in high-impact journals, encompassing original articles, case reports, technical notes, reviews, commentaries and editorials. From these 1,394 papers, at least 90 comprised original studies containing patient data. A total of 48% of the articles housed in the institutional digital library of academic papers are accessible through open access. FMUSP-HC has actively encouraged researchers to submit their work for publication in open access journals, reflecting a strategic alignment with the broader movement toward open science.

### Implementation challenges

The COVID-19 Steering Committee and the research management team faced several implementation challenges during the course of their work at HC-FMUSP. These challenges, their possible causes and the ways by which they were dealt with are discussed in the sub-items below and summarized in Table 2.

**Table 2:** Challenges identified during the implementation of institutional research initiatives at HC-FMUSP in the COVID-19 pandemic.

Challenges	Barriers	Actions taken
How to disseminate information about the initiatives within the HC-FMUSP system	Fragmented institutional communication	Frequent and detailed internal communication about the initiatives (one-on-one discussions with research leaders, presentations to groups and internal collegiate, repeated electronic memos to mailing lists, progress reports to participating research groups) Building of an open website (https://sites.google.com/view/covid-19-hcfmusp)
How to overcome conflicts regarding leadership, ownership of information and data sharing	Habits of research groups to work either individually or with a few trusted partners	Identification of (and frequent communication with) a few experienced and respected HC-FMUSP researchers willing to manage key components of the institutional collaborative research initiatives Transparent alignment with those leaders to guide the uniform implementation of actions fostering maximal inclusion of (and cooperation between) potentially interested research groups Stressing of the principles of inclusion, transparency and cooperativeness in all communications with research groups Access to data from the REDCap databases provided solely by the direct management team, after swift authorization of the COVID-19 Steering Committee using objective and previously advertised criteria
How to award fair credit and co-authorship opportunities in publications	Frustration of professionals that might not have opportunities to exercise their research interests due to overload of clinical and management work during the pandemic Risk of honorary authorship	Use of a corporate coauthor including members selected according to objective criteria (i.e., professionals who helped significantly in the construction of the hospital databases). Setting of rules whereby individuals from the corporate coauthor were invited to contribute intellectually to (and approve the final version of) original papers that used data from significant numbers (>800) of patients For other key professionals from the crisis committee who had no familiarity with or interests in research on clinical aspects of COVID-19, use of a second corporate name cited in the Acknowledgements sections of manuscripts. Some of those individuals were also invited to contribute intellectually to specific papers evaluating non-clinical aspects of COVID-19 Avoidance of inclusion of coauthors that did not meet the criteria of the International Committee of Medical Journal Editors. Professors whose leadership was restricted to research administration of the initiatives were listed as authors only in specific cases when they did take part in the planning of investigations and analyses, interpretation of results and writing-up of manuscripts
How to facilitate the hands-on use of institutional databases by researchers	Lack of familiarity of the research groups with the databases’ structure	Strengthening of the role of the direct research managing team shown in Figure 1 in the overseeing of the research teams that worked on analyses using the databases. The management team helped researchers to select data fields relevant to their study goals and to understand how those elements were coded in the databases, as well as working to prevent unnecessary duplication of analyses by different groups, errors in the interpretation of numbers and variables, and discrepancies when similar data was reported across separate papers
How to balance the choice of instruments for the multidisciplinary follow-up assessments of patients	Multiplicity of interests of different research groups Risk of duplication/overlap of information collected using different scales Risk that questions proposed by some research groups would not be valued as equally meritorious by other teams	Validation by the COVID-19 Steering Committee of the direct management team as the mediator in the negotiations between research groups, in order to ensure that the assessment battery would be as thorough as possible without overburdening research participants Democratic mediation of decisions, in order to facilitate the inclusion of the largest possible number of research groups with diverse interests
How to deal with ethical issues regarding use of data from hospitalized patients with COVID-19	Risk of privacy breaches, re-identification and misuse of data extracted from medical records Difficulties to obtain informed consent from hospitalized patients or their family members for storing blood samples in the biobank	Recruitment of a team of medical students to seek informed consent during in-hospital stay from patients and family members for storing leftover blood from diagnostic tests Use of the follow-up visit conducted months after hospitalization to retrospectively obtain permission for use of leftover blood from diagnostic tests stored during hospitalization Request for the Brazilian National Research Ethics Committee to grant permission for the scientific use of de-identified healthcare data and biological materials from patients deceased due to COVID-19, considering the invaluable importance of medical research during the extraordinary pandemic circumstances
How to minimize delays in the dispensing and use of blood samples from the biobank	Lack of previous experience of the management teams Overload of the management teams with work on the other fronts of data organization Difficulties of potentially interested research groups to allocate funds to cover costs of processing/analyzing samples	Top-down orientation for individual research groups to work collaborative in the sharing of costs whenever possible Submission of a multidisciplinary grant proposal to FAPESP in order to raise further funds for large-scale analyses of samples Expansion of opportunities for experienced groups outside the HC-FMUSP system to get access to blood samples for collaborative studies

FAPESP, Fundação de Amparo à Pesquisa do Estado de São Paulo (São Paulo Research Foundation); HC-FMUSP, Hospital das Clínicas da Faculdade de Medicina da Universidade de São Paulo; REDCap, Research Electronic Data Capture.

#### Dissemination of information and questions regarding leadership

Large academic health system complexes like HC-FMUSP often face communication challenges, hindering the dissemination of information about institutional initiatives (22, 67). During the pandemic, our research management teams encountered difficulties in reaching all potentially interested research groups. Additionally, the shift toward a more collaborative research approach was met with hesitation by the HC-FMUSP scientific community, leading to concerns about conflicts over leadership and data ownership.

To address these challenges, we appointed experienced and respected HC-FMUSP researchers to lead different components of the institutional initiatives. We maintained frequent communication with these leaders to make strategic decisions and ensure consistent implementation of actions with transparency and cooperation. Several channels of communication were used to increase overall institutional awareness about the principles of inclusion, transparency, and cooperation of the initiatives, through small-group discussions, sharing of presentations, and sending of memos and progress reports. This process was time-consuming but rewarding, since approximately 23% of the total number of research groups of HC-FMUSP (50 out of 220) eventually agreed to participate in the collaborative initiatives. Over time, open communication appeared to reduce the number of conflicts regarding leadership, data ownership, and data sharing that had initially arisen.

Despite our efforts to improve communication and promote collaboration among research groups at HC-FMUSP, some expressed dissatisfaction with their level of inclusion and access to information. To address this, we launched a website for the institutional initiatives.^3^ While this initiative was implemented relatively late in the process (September 2022), we hope that it will serve as a valuable resource for all interested parties, providing updates on progress, relevant publications, and opportunities for involvement in ongoing research activities.

#### Questions about credit and authorship

A second challenge we faced was how to give credit to the healthcare professionals and management teams of HC-FMUSP whose contributions were essential in creating the institutional databases discussed in this paper. A corporate coauthor, the *HC-FMUSP COVID-19 Study Group*, was created to acknowledge their contributions, and grant them opportunities to have some degree of intellectual involvement in research activities. This group, consisting of 31 professionals, was selected based on objective criteria. We set a rule whereby those individuals would be invited to contribute intellectually to (and approve the final version of) all the original scientific publications that would be based on data collected from significant amounts (>800) of patients from the institutional databases. The goal was to acknowledge individuals that did not take part in the conception and writing of the first draft of articles, but who helped significantly in the construction of the databases. The use of such corporate coauthor gave the opportunity for its members to contribute intellectually to the interpretation of the results and finalization of several manuscripts.

The contributions of other key professionals from the crisis committee who had no familiarity or interests in research on clinical aspects of COVID-19 was acknowledge by the creation of a second corporate name (*HC-FMUSP COVID-19 task force*), quoted in the Acknowledgements sections of manuscripts, listing individuals and the key contributions within the group involved in overall infrastructure and logistics during the pandemic. Some of those individuals were also invited to contribute intellectually to (and therefore were included as individual authors in) a few specific manuscripts evaluating non-clinical aspects of COVID-19, e.g., costs of care (44).

An associated challenge that emerged concerned the risk of professors involved in the management of the initiatives being offered honorary co-authorship in any papers that utilized the institutional databases, simply based on their administrative leadership. This situation was deemed unacceptable as per the guidelines recommended by the International Committee of Medical Journal Editors (68), and also went against the principle of true cooperativeness that our institution aimed to bring to the initiatives. Therefore, the professors whose leadership was restricted to research administration of the initiatives were neither included as members of the corporate coauthor cited above (*HC-FMUSP COVID-19 Study Group*) nor individually named in the list of authors of papers (except in situations in which those leaders did play an intellectual role in the planning of investigations and analyses, interpretation of results and writing-up of manuscripts).

#### Lack of familiarity with and/or difficulties understanding the databases’ structure

A third challenge faced by our group was that some research groups presented a degree of unfamiliarity with and/or difficulties to understand the databases’ structure, the clinical case definitions based on key variables for symptom-based, laboratory and radiological diagnoses (34, 35), and the structured flow for inclusion and exclusion of cases (36). To address this, the research managing team (Figure 1) played a constant role in overseeing and assisting research teams. Through repeated interactions, the managing team developed domain-expertise, gaining a deep understanding of the database structure and variables, and ultimately helping researchers in the selection of relevant data fields and comprehension of how the data was coded within the database. This domain-expertise facilitated the application of data to healthcare problems and research questions (10, 69), preventing duplication of analyses, interpretation errors, and discrepancies in reported data across papers.

#### Choice of instruments and examinations to be included in the multidisciplinary follow-up assessment battery

A fourth challenge involved negotiations among research groups with different interests to determine the scope of the multidisciplinary follow-up assessments. The goal was to ensure a comprehensive assessment while avoiding to burden the participants. Compromises were made to prevent overlap and reach consensus on assessment instruments. The leadership (Figure 1) mediated these negotiations, aiming to democratize access to the program for diverse research groups, and most research groups demonstrated flexibility and a willingness to compromise.

#### Participants’ consent

A most relevant challenge faced over the course of the institutional initiatives described herein regarded patient consent. Our overall approach was carefully planned to avoid privacy breaches, re-identification, and misuse of data extracted from medical files. The Good Clinical Practice (GCP) guidelines (70) were followed to guarantee ethical and scientific quality standards in the conduction of the studies.

Obtaining patient or family members consent for the COVID-19 biobank data was a complex and time-consuming process due to the conditions of hospital strain and strict isolation needs. The follow-up visits conducted months after hospitalization were highly valuable to retrospectively obtain permission for use of leftover blood from diagnostic tests stored during hospitalization. The use of blood samples from surviving patients for which we had not obtained consent was strictly forbidden. For deceased patients, the prospect of posthumously using their biological samples for research was discussed with the next-of-kin whenever possible, in order to obtain consent. Nonetheless, during the unusual and hectic circumstances of the COVID-19 pandemic, identifying and successfully contacting deceased patients’ relatives was often not realistic. Considering the invaluable importance of medical research during the pandemic, and to prevent further loss of human life, the Brazilian National Research Ethics Committee granted permission for de-identified healthcare data and biological materials from patients deceased due to COVID-19 to be used by research groups in their ethically approved research projects, even without patient consent.

#### Difficulties in the dispensing of blood samples from the biobank

A final challenge was the imbalance between the large amount of stored blood samples and the slow rate of dispensing of such biological material for use in research studies. The research management teams were unprepared for this initiative (given its novelty) and burdened with work at the two other fronts of data organization, causing delays in advertising the biobank and providing samples to approved studies. This frustrated research groups at HC-FMUSP eager to utilize the material promptly. Some interested groups also faced financial constraints for sample processing and analysis. However, the recently approved grant by FAPESP has secured funds for biomarker investigations, benefiting from the stored samples. Furthermore, we have expanded collaboration opportunities with external research groups (both from Brazil and abroad), offering access to samples and clinical data for joint investigations. Research collaboration agreements are currently under preparation, whereby we will share both blood samples and clinical data for additional investigations in collaboration with those groups. These measures will help to increase the pace of sample dispensing and facilitate research using the valuable resources of the biobank.

### Recommended strategies to enhance collaboration within research institutions

Based on the lessons learned from the actions described in this paper, combined with previous literature (10, 16, 22, 69, 71–73), we present in Table 3 a set of recommendations for strategies aimed at enhancing collaboration within research institutions. The rationale behind these recommendations is to foster collaborations, complementing rather than replacing traditional research. All of the strategies summarized in Table 3 were fully or partially implemented in our institutional approach.

**Table 3:** Strategies recommended for enhancing collaboration within research institutions, based on lessons learned and previous literature.

Recommendations		Discussion
1	Start by creating a clearly defined governance board	The defined governance board will be responsible for: 1. establishing policies and guidelines for data collection, documentation, storage, retention, and sharing, ensuring that the data is appropriately managed throughout the entire research lifecycle; 2. establishing policies to ensure compliance with ethical guidelines and with relevant data security regulations (safeguarding data against unauthorized access, breaches, and data loss); 3. establishing clearly defined protocols and mediating the sharing of resources, such as equipment, data, facilities, or funding, to support collaborative research; 4. mapping, monitoring, and supporting the efforts of research groups to produce research papers; 5. in the domain of health research, this board could take on the responsibility of identifying common/standardized measures for health conditions that would benefit a majority of, or all, the institution’s researchers; and any measures necessary for specific studies of higher interest at any given time. That could lead to the proposition of strategies to foster cross-disciplinary studies of institutional, national or global interest. The leadership positions could be assigned through institutional allocation or determined by votes from the research community.
2	Build infrastructure and resources	Institutions should allocate adequate funding to support research, investing in robust research organizational infrastructure, encompassing both the physical structures and systems as well as the underlying support personnel necessary for institutionally managed research collaborations.
3	Establish diverse multidisciplinary research teams	Complex research challenges require expertise from various disciplines. By creating diverse multidisciplinary research teams, institutions can leverage different perspectives and knowledge to address various research questions.
4	Establish clearly defined roles and responsibilities	Clearly define roles, responsibilities, and expectations for each collaborator involved in the research collaboration. Establishing a framework for decision-making, task allocation, and accountability helps prevent misunderstandings and ensures that everyone knows their contribution and commitment to the collaboration.
5	Create a team of professional data analytics and data science experts	To effectively handle large datasets and/or datasets that involve the integration of secondary data (such as data extracted from electronic health records), it is important to create a dedicated team of professional data analytics and data science experts to ensure that data is accurate, consistent, and reliable. The team should develop robust processes to prevent data errors, duplicates, and inconsistencies, resulting in improved data quality and integrity. Additionally, they should streamline data integration, standardization, and harmonization across various systems and departments. The team’s responsibilities also encompass managing data throughout its lifecycle, including identifying and mitigating security risks, ensuring data protection and compliance, and providing necessary technical assistance and support.
6	Establish a proficient hands-on research managing team	A hands-on research managing team, possessing a comprehensive understanding of the data and of the institutional research goals, will assist researchers in selecting relevant data fields and facilitate the application of data to their research questions, thereby avoiding redundant analyses, interpretation errors, and inconsistencies in reported data across various papers
7	Establish effective communication	Establish open lines of communication to facilitate information-sharing, exchange of ideas, and updates on progress. Clear and frequent communication helps build trust, resolve conflicts, and keep all collaborators engaged and informed. Innovative methods could be used; e.g. technology tools such as collaboration platforms or websites to present results and updates; or chatbots with 24/7 availability to provide quick and accurate responses to common queries, saving time for employees and reducing the burden on human resources by assisting with frequently asked questions, policy inquiries, or providing access to relevant documents and resources (complementing human communication rather than replacing it). It is also vital to establish feedback mechanisms by creating channels for employees to provide feedback, suggestions, and concerns. This can be through surveys, suggestion boxes, or regular feedback sessions.
8	Build trust and respect among collaborators, focusing on cooperation, equity, and transparency	Foster an environment of trust, respect, and integrity among collaborators. Encourage open and honest discussions, acknowledge diverse perspectives, and value each collaborator’s contributions. Trust is essential for sharing resources, data, and research findings.
9	Ensure mutual benefit for all parts involved, emphasizing meaningful outcomes	Ensure that all parties involved in the research collaboration can derive benefits from the partnership. Identify how each collaborator’s expertise, resources, or access to data can contribute to the collaborative effort, creating an advantageous situation for all. In the context of healthcare research, collaborative research must also always be carried out within a voluntary participation scenario governed by values of reciprocity with and non-exploitation of the patients and service of the public good.
10	Prioritize the timely sharing and dissemination of research findings	Request that collaborators swiftly publish their work in reputable scientific journals to make it accessible to the broader scientific community. Additionally, institutions can organize meetings, conferences, symposiums, and workshops to facilitate the exchange of knowledge and promote dialog among researchers.
11	Strengthen national and international research collaborations	Data from a single research center are less relevant than data collected from multiple centers; therefore, it is crucial to incorporate institutional data into a broader research network.

## Discussion

Based on the relatively large size of the databases that the HC-FMUSP teams were able to compile, the institutional initiatives described in the present paper may be judged as successful in their aim to foster productive, large-scale research. These initiatives captured demographic and clinical data from thousands of COVID-19 cases treated in a densely urbanized region from a low-and-middle-income country (LMIC), organized in interconnected REDCap databases, available for investigations by over 30 research groups so far. Additionally, follow-up data from hundreds of COVID-19 patients, assessed 6 to 11 months after hospitalization through comprehensive in-person evaluations, have been used by more than 20 research groups. Finally, thousands of blood samples collected during the acute phase and follow-up assessments remain stored for future investigations. Most papers published within this initiative were interdisciplinary, with an unprecedented level of interaction between internal groups that had not previously worked together. To our knowledge, this was the first large-sized collaborative experience of such kind inside an academic hospital complex in Brazil.

Innovative strategies, different from traditional clinical research methods, are necessary to drive advances into the healthcare field and further improve public health (5). Implementing collaborative research management models offers several advantages, including pooling diverse knowledge, enhancing research productivity, cross-disciplinary fertilization, and improved access to expertise, equipment, and funds (7). The extraordinary context of the COVID-19 pandemic confirmed the notion that complex human health problems demand innovative and collaborative solutions combining knowledge from different scientific disciplines (2, 3, 7). Additionally, the pandemic has emphasized the decisive role of data sharing and open access to scientific publications in expediting scientific advancement with efficiency. During the global health crisis, journals and publishers responded by unlocking access to their content and by promoting a marked decrease in the time required for article publication. Furthermore, there has been a surge in the release of preprints, albeit without formal peer review. While these initiatives have accelerated the pace of scientific communication, they have concurrently evidenced the essential need for rigor in the scientific community. While not a fit-for-all solution, large-scale cross-disciplinary research management models, like the one described in this paper, can foster collaboration, reduce inefficiency, and produce high-quality, large-scale research results (20).

While over thirty studies on COVID-19 have been published in peer-reviewed journals using institutional databases (30, 31, 33, 36–66), contributing significant data to the existing literature, there was a considerable delay in their production, with most being accepted for publication in late 2021, or later. The delay in publishing findings from institutional databases can be attributed to various factors such as research groups being involved in completing their own studies and others being overwhelmed with healthcare and teaching activities during the pandemic. However, the major cause of the publication delay was the time required for organizing this process in our institution. Additionally, the high submission rate of COVID-19-related manuscripts from different parts of the world to highly-ranked peer-reviewed journals possibly led to an increased level of competitiveness, resulting in a higher threshold for acceptance of papers by those journals. Nonetheless, we are optimistic about the future of the program as we consolidate the data, establish the biobank, and receive grant support, which will ensure a more robust and sustainable program.

Regarding the multidisciplinary follow-up initiative, the COVID-19 Steering Committee encouraged participating groups to publish interim findings [e.g., (58)]. However, most teams opted to wait until data collection was completed in April 2021. By that time, several observational studies on long COVID had already been published by research groups from China, Europe, and the United States [e.g., (74–77)], and that led to some of our manuscripts being rejected by high-profile journals on the grounds of lack of novelty.

For the blood samples from the COVID-19 biobank, there were difficulties and delays in dispensing aliquots, which may explain why only two studies have been published to date using this biological material. Nevertheless, our collection of biological material is still regarded as highly precious, as it was obtained from a large sample of unvaccinated COVID-19 patients for whom we have also comprehensive data both about the acute disease and follow-up assessments. This explains the current interest raised by external research groups both from Brazil and abroad in using such databases in further collaborative research studies with HC-FMUSP teams. Moreover, a new wave of biomarker investigations by HC-FMUSP groups is expected to take place thanks to the funds that have been recently secured through the large grant approved by FAPESP.

Efforts to foster large-scale data-driven research require multidisciplinary collaboration, crossing the boundaries of healthcare, with additional teams required with skills spanning statistics, computational systems and data science (6, 78, 79). Implementation of EHR brings healthcare closer to data science, computational biology, and artificial intelligence (10). In our initiatives, we applied artificial intelligence and contemporary computational methods to analyze hospitalization data through collaborations with computer science groups (30, 48, 50). Caution is advised regarding such secondary uses of healthcare data from EHR due to potential misinterpretation and concerns about data quality, especially missing or inaccurate data (72). Nevertheless, routine healthcare data, i.e., data generated from routine, standard care of patients, may be a particularly valuable source to inform treatment decisions, because it better represents the real-world uncontrolled conditions faced in clinical practice.

Albeit large, our COVID-19 hospitalization databases were substantially more modest in size compared to initiatives conducted in other settings using EHR. While we collected data from thousands of patients during hospital stays and hundreds of follow-up assessments, studies in other countries have included hundreds of thousands or millions of subjects [e.g., (80–86)]. However, our institutional approach combining different sources of data and involving several teams working in collaboration improved the quantity and quality of the health data obtained from each subject. This led to the construction of comprehensive institutional databases from a representative cohort of subjects from a large LMIC city, with information on complex patients with multi-morbidity and polypharmacy, and who were treated in a real-world setting. These databases include detailed information for subjects from racial-ethnic minorities, socioeconomically disadvantaged, and other underprivileged or discriminated-against populations, who continue to experience a disproportionate share of many acute or chronic diseases and adverse health outcomes (9, 87, 88). Despite all the limitations and challenges, the implemented collaborative research actions resulted in one of the largest severe COVID-19 cohorts with in-person follow-up multidisciplinary evaluations to date.

As it appears to be the norm in most universities (16, 22, 67), the different research groups at HC-FMUSP distinguish themselves by their varied areas of interest, assumptions, priorities, methods, and research practices. These structural and cultural differences between disciplines may constitute significant barriers to collaborative research, and that was a difficulty faced during the implementation of our institutional collaborative COVID-19 research approach. It is not uncommon for talented, high-performing research leaders to find collaboration unnatural, after years working to set themselves apart and propel their academic careers (89). Up until now, there is limited research that explicitly examines how to encourage collaboration in settings similar to the HC-FMUSP system (16, 22, 73). Additional studies are necessary to increase understanding on how to further help researchers to overcome barriers and lean toward more collaborative science. Institutional initiatives such as the one described herein should be evaluated using qualitative survey methods, in order to investigate the perceptions of members of the research community about the proposed management approach and the challenges faced during its implementation.

## Conclusion

Several experts have predicted that we are moving toward an era of research where openly shared data will become the norm (5, 10, 23, 90, 91). The results obtained from shared knowledge and discovery diminish the importance of securing intellectual property of healthcare data (without forgoing patient’s privacy) (90, 91). Consequently, independent research might become less sustainable than collaborative research. Thus, researchers are beginning to prepare for a future when science will be led by those who have the resources and skills to exploit knowledge assets fastest, rather than by those who own it (23). In this context, scientific collaboration provides a highly effective means to produce knowledge by allowing the sharing of skills, expertise and resources (5, 15).

Research is a crucial component of an effective epidemic response, and the preparation process should include a well-defined plan for organizing and sharing data. This aspect is just as important as all other elements of the response. Although a single model may not be appropriate for all contexts, cross-disciplinary collaboration should make health research systems more efficient to generate the best evidence (5). The top-down collaborative model implemented at HC-FMUSP during the COVID-19 pandemic has the aspiration to motivate a broader use of such kind of institutional approach to enable further scientific developments, helping to transform health care and improve human health. Our current COVID-19 databases may serve as prototypes for the development of additional databases addressing other areas of clinical interest. Such large-scale databases are likely to grow more rapidly, be more complete and be more useful if the three following conditions are met: universal use of automatically-extracted electronic health records; a greater acceptance of cross-disciplinary collaboration; and the cultivation of a culture of more open data sharing.

## Data availability statement

Publicly available datasets were analyzed in this study. This data can be found at: Data available in our institutional databases mentioned in this paper can be accessed for COVID-related research with (1) a study protocol approved by a research ethics committee and (2) a Data Use Request. Data access instructions can be found at: https://sites.google.com/view/covid-19-hcfmusp.

## Ethics statement

The studies involving humans were approved by CAPPesq – Comissão de Ética para Análise de Projetos de Pesquisa. The studies were conducted in accordance with the local legislation and institutional requirements. Written informed consent for participation in this study was provided by the participants’ legal guardians/next of kin.

## Author contributions

AR: Conceptualization, Investigation, Writing – original draft, Writing – review & editing. AA: Investigation, Writing – review & editing. CC: Investigation, Project administration, Writing – review & editing. HS: Investigation, Writing – review & editing. PF: Funding acquisition, Project administration, Resources, Writing – review & editing. VS: Funding acquisition, Project administration, Resources, Writing – review & editing. MG: Investigation, Project administration, Writing – review & editing. LK: Investigation, Project administration, Writing – review & editing. EK: Writing – review & editing. AP: Investigation, Writing – review & editing. VC: Data curation, Formal analysis, Investigation, Writing – review & editing. KS: Data curation, Formal analysis, Investigation, Writing – review & editing. EA: Data curation, Methodology, Writing – review & editing. AS: Investigation, Writing – review & editing. ES: Investigation, Writing – review & editing. UR: Investigation, Writing – review & editing. RF: Investigation, Writing – review & editing. AM-M: Investigation, Writing – review & editing. AL: Investigation, Writing – review & editing. MSa: Investigation, Writing – review & editing. JF: Investigation, Writing – review & editing. CS: Investigation, Writing – review & editing. TM: Investigation, Writing – review & editing. NG: Investigation, Writing – review & editing. LL: Investigation, Writing – review & editing. MB: Investigation, Writing – review & editing. LB: Investigation, Writing – review & editing. AD: Investigation, Writing – review & editing. MSe: Investigation, Writing – review & editing. JM: Investigation, Writing – review & editing. OF: Investigation, Writing – review & editing. VR: Investigation, Writing – review & editing. MM-C: Investigation, Writing – review & editing. SC: Investigation, Writing – review & editing. GC: Investigation, Writing – review & editing. EB: Investigation, Writing – review & editing. RC: Investigation, Writing – review & editing. TB: Investigation, Writing – review & editing. GB: Conceptualization, Investigation, Methodology, Supervision, Writing – original draft, Writing – review & editing.

## Footnotes

^1^#HCCOMVIDA initiative; https://www.viralcure.org/hc.

^2^ https://observatorio.fm.usp.br/handle/OPI/42970

^3^ https://sites.google.com/view/covid-19-hcfmusp

## References

[1] Tenforde MW Billig Rose E Lindsell CJ Shapiro NI Files DC Gibbs KW. Characteristics of adult outpatients and inpatients with COVID-19 - 11 academic medical centers, United States, march-may 2020. MMWR Morb Mortal Wkly Rep. (2020) 69:841–6. doi: 10.15585/mmwr.mm6926e3, PMID: 32614810 PMC7332092

[2] Nalbandian A Sehgal K Gupta A Madhavan MV McGroder C Stevens JS. Post-acute COVID-19 syndrome. Nat Med. (2021) 27:601–15. doi: 10.1038/s41591-021-01283-z, PMID: 33753937 PMC8893149

[3] Del Rio C Collins LF Malani P. Long-term health consequences of COVID-19. JAMA. (2020) 324:1723–4. doi: 10.1001/jama.2020.19719, PMID: 33031513 PMC8019677

[4] Chan AW Song F Vickers A Jefferson T Dickersin K Gotzsche PC. Increasing value and reducing waste: addressing inaccessible research. Lancet. (2014) 383:257–66. doi: 10.1016/S0140-6736(13)62296-5, PMID: 24411650 PMC4533904

[5] Subbiah V. The next generation of evidence-based medicine. Nat Med. (2023) 29:49–58. doi: 10.1038/s41591-022-02160-z 36646803

[6] Bennett TD Callahan TJ Feinstein JA Ghosh D Lakhani SA Spaeder MC. Data science for child health. J Pediatr. (2019) 208:12–22. doi: 10.1016/j.jpeds.2018.12.041, PMID: 30686480 PMC6486872

[7] van Rijnsoever FJ Hessels LK. Factors associated with disciplinary and interdisciplinary research collaboration. Res Policy. (2011) 40:463–72. doi: 10.1016/j.respol.2010.11.001

[8] Ioannidis JP Greenland S Hlatky MA Khoury MJ Macleod MR Moher D. Increasing value and reducing waste in research design, conduct, and analysis. Lancet. (2014) 383:166–75. doi: 10.1016/S0140-6736(13)62227-8, PMID: 24411645 PMC4697939

[9] Zhang X Perez-Stable EJ Bourne PE Peprah E Duru OK Breen N. Big data science: opportunities and challenges to address minority health and health disparities in the 21st century. Ethn Dis. (2017) 27:95–106. doi: 10.18865/ed.27.2.95, PMID: 28439179 PMC5398183

[10] Sanchez-Pinto LN Luo Y Churpek MM. Big data and data science in critical care. Chest. (2018) 154:1239–48. doi: 10.1016/j.chest.2018.04.037, PMID: 29752973 PMC6224705

[11] Ioannidis JP. How to make more published research true. PLoS Med. (2014) 11:e1001747. doi: 10.1371/journal.pmed.1001747 25334033 PMC4204808

[12] Stokols D Fuqua J Gress J Harvey R Phillips K Baezconde-Garbanati L. Evaluating transdisciplinary science. Nicotine Tob Res. (2003) 5:21–39. doi: 10.1080/14622200310001625555 14668085

[13] Klein JT. Evaluation of interdisciplinary and transdisciplinary research: a literature review. Am J Prev Med. (2008) 35:S116–23. doi: 10.1016/j.amepre.2008.05.010 18619391

[14] Lyall C Meagher L Bruce A. A rose by any other name? Transdisciplinarity in the context of UK research policy. Futures. (2015) 65:150–62. doi: 10.1016/j.futures.2014.08.009

[15] Ynalvez MA Shrum WM. Professional networks, scientific collaboration, and publication productivity in resource-constrained research institutions in a developing country. Res Policy. (2011) 40:204–16. doi: 10.1016/j.respol.2010.10.004

[16] Urton D Murray D. Project manager's perspectives on enhancing collaboration in multidisciplinary environmental management projects. Project Leaders Soc. (2021) 2:100008. doi: 10.1016/j.plas.2021.100008

[17] Miethke-Morais A Perondi B Harima L Montal AC Baldassare RM Moraes DP. Overcoming barriers to providing comprehensive inpatient care during the COVID-19 pandemic. Clinics (Sao Paulo). (2020) 75:e2100. doi: 10.6061/clinics/2020/e2100, PMID: 32609227 PMC7314577

[18] Perondi B Miethke-Morais A Montal AC Harima L Segurado AC Hospital das Clinicas COVID-19 Crisis Management Committee. Setting up hospital care provision to patients with COVID-19: lessons learnt at a 2400-bed academic tertiary center in Sao Paulo, Brazil. Braz J Infect Dis. (2020) 24:570–4. doi: 10.1016/j.bjid.2020.09.005, PMID: 33157034 PMC7604059

[19] Busatto GF Silva CA Pereira AJR Bonfa E Barros-Filho TEP. Scientific legacy of COVID-19 at the FMUSP-HC academic health system: current status and implications for the future. Clinics (Sao Paulo). (2021) 76:e3630. doi: 10.6061/clinics/2021/e3630, PMID: 34909915 PMC8612300

[20] Al-Shahi Salman R Beller E Kagan J Hemminki E Phillips RS Savulescu J. Increasing value and reducing waste in biomedical research regulation and management. Lancet. (2014) 383:176–85. doi: 10.1016/S0140-6736(13)62297-7, PMID: 24411646 PMC3952153

[21] Bond-Barnard TJ Fletcher L Steyn H. Linking trust and collaboration in project teams to project management success. Int J Manag Proj Bus. (2018) 11:432–57. doi: 10.1108/IJMPB-06-2017-0068

[22] Crooks CV Exner-Cortens D Siebold W Moore K Grassgreen L Owen P. The role of relationships in collaborative partnership success: lessons from the Alaska fourth R project. Eval Program Plann. (2018) 67:97–104. doi: 10.1016/j.evalprogplan.2017.12.007, PMID: 29289925

[23] Adams J. Collaborations: the fourth age of research. Nature. (2013) 497:557–60. doi: 10.1038/497557a 23719446

[24] World Health Organization. WHO global clinical platform for COVID-19: Core case report form (CRF), version 8 April 2020, revised 13 July 2020, revised 29 November 2021. Geneva: World Health Organization (2021).

[25] Harris PA Taylor R Thielke R Payne J Gonzalez N Conde JG. Research electronic data capture (REDCap)—a metadata-driven methodology and workflow process for providing translational research informatics support. J Biomed Inform. (2009) 42:377–81. doi: 10.1016/j.jbi.2008.08.010, PMID: 18929686 PMC2700030

[26] Busatto GF De Araujo AL Duarte AJDS Levin AS Guedes BF Kallas EG. Post-acute sequelae of SARS-CoV-2 infection (PASC): a protocol for a multidisciplinary prospective observational evaluation of a cohort of patients surviving hospitalisation in Sao Paulo, Brazil. BMJ Open. (2021) 11:e051706. doi: 10.1136/bmjopen-2021-051706, PMID: 34193506 PMC8249176

[27] Macedo BR Garcia MVF Garcia ML Volpe M Sousa MLA Amaral TF. Implementation of tele-ICU during the COVID-19 pandemic. J Bras Pneumol. (2021) 47:e20200545. doi: 10.36416/1806-3756/e20200545, PMID: 33950091 PMC8332846

[28] Scudeller PG Lamas CA Alvarenga AM Garcia ML Amaral TF de Oliveira MR. Tele-intensive care unit program in Brazil: implementation and expansion. Telemed Rep. (2023) 4:109–17. doi: 10.1089/tmr.2023.0017, PMID: 37283854 PMC10240323

[29] Scudeller PG Pereira AJ Cerri GG Jatene FB Bego M Amaral TF. Telemedicine in Brazil: teleconsultations at the largest University Hospital in the Country. Telemed Rep. (2023) 4:193–203. doi: 10.1089/tmr.2023.0012, PMID: 37529769 PMC10389256

[30] Carvalho CRR Chate RC Sawamura MVY Garcia ML Lamas CA Cardenas DAC. Chronic lung lesions in COVID-19 survivors: predictive clinical model. BMJ Open. (2022) 12:e059110. doi: 10.1136/bmjopen-2021-059110, PMID: 35697456 PMC9195157

[31] Carvalho CRR Lamas CA Chate RC Salge JM Sawamura MVY de Albuquerque ALP. Long-term respiratory follow-up of ICU hospitalized COVID-19 patients: prospective cohort study. PLoS One. (2023) 18:e0280567. doi: 10.1371/journal.pone.0280567, PMID: 36662879 PMC9858876

[32] Ahmed M Advani S Moreira A Zoretic S Martinez J Chorath K. Multisystem inflammatory syndrome in children: a systematic review. EClinicalMedicine. (2020) 26:100527. doi: 10.1016/j.eclinm.2020.100527, PMID: 32923992 PMC7473262

[33] Fink TT Marques HHS Gualano B Lindoso L Bain V Astley C. Persistent symptoms and decreased health-related quality of life after symptomatic pediatric COVID-19: a prospective study in a Latin American tertiary hospital. Clinics (Sao Paulo). (2021) 76:e3511. doi: 10.6061/clinics/2021/e3511, PMID: 34852145 PMC8595593

[34] Corman VM Landt O Kaiser M Molenkamp R Meijer A Chu DK. Detection of 2019 novel coronavirus (2019-nCoV) by real-time RT-PCR. Euro Surveill. (2020) 25:45. doi: 10.2807/1560-7917.ES.2020.25.3.2000045, PMID: 31992387 PMC6988269

[35] Lisboa Bastos M Tavaziva G Abidi SK Campbell JR Haraoui LP Johnston JC. Diagnostic accuracy of serological tests for covid-19: systematic review and meta-analysis. BMJ. (2020) 370:m2516. doi: 10.1136/bmj.m2516 32611558 PMC7327913

[36] Menezes MCS Santinelli Pestana DV Ferreira JC Ribeiro de Carvalho CR Felix MC Marcilio IO. Distinct outcomes in COVID-19 patients with positive or negative RT-PCR test. Viruses. (2022) 14:175. doi: 10.3390/v14020175 35215772 PMC8874612

[37] Ferreira JC Moreira TCL de Araujo AL Imamura M Damiano RF Garcia ML. Clinical, sociodemographic and environmental factors impact post-COVID-19 syndrome. J Glob Health. (2022) 12:05029. doi: 10.7189/jogh.12.05029, PMID: 35939273 PMC9359428

[38] Aliberti MJR Szlejf C Avelino-Silva VI Suemoto CK Apolinario D Dias MB. COVID-19 is not over and age is not enough: using frailty for prognostication in hospitalized patients. J Am Geriatr Soc. (2021) 69:1116–27. doi: 10.1111/jgs.17146, PMID: 33818759 PMC8251205

[39] Ferreira JC Ho YL Besen BAMP Malbouisson LMS Taniguchi LU Mendes PV. Protective ventilation and outcomes of critically ill patients with COVID-19: a cohort study. Ann Intensive Care. (2021) 11:92. doi: 10.1186/s13613-021-00882-w, PMID: 34097145 PMC8182738

[40] Gil S Jacob Filho W Shinjo SK Ferriolli E Busse AL Avelino-Silva TJ. Muscle strength and muscle mass as predictors of hospital length of stay in patients with moderate to severe COVID-19: a prospective observational study. J Cachexia Sarcopenia Muscle. (2021) 12:1871–8. doi: 10.1002/jcsm.12789 34523262 PMC8661522

[41] Gonçalves FAR Besen BAMP Lima CA Cora AP Pereira AJR Perazzio SF. Use and misuse of biomarkers and the role of D-dimer and C-reactive protein in the management of COVID-19: a post-hoc analysis of a prospective cohort study. Clinics (Sao Paulo). (2021) 76:e3547. doi: 10.6061/clinics/2021/e3547, PMID: 34909913 PMC8612302

[42] Maeda MFY Brizot ML Gibelli MABC Ibidi SM Carvalho WB Hoshida MS. Vertical transmission of SARS-CoV2 during pregnancy: a high-risk cohort. Prenat Diagn. (2021) 41:998–1008. doi: 10.1002/pd.5980, PMID: 34101871 PMC8242902

[43] Marques HHS Pereira MFB Santos ACD Fink TT Paula CSY Litvinov N. Differences in children and adolescents with SARS-CoV-2 infection: a cohort study in a Brazilian tertiary referral hospital. Clinics (Sao Paulo). (2021) 76:e3488. doi: 10.6061/clinics/2021/e3488, PMID: 34852143 PMC8595603

[44] Miethke-Morais A Cassenote A Piva H Tokunaga E Cobello V Rodrigues Goncalves FA. COVID-19-related hospital cost-outcome analysis: the impact of clinical and demographic factors. Braz J Infect Dis. (2021) 25:101609. doi: 10.1016/j.bjid.2021.101609, PMID: 34454894 PMC8373618

[45] Baptista FS Paganoti CF Gomez UT Peres SV Malbouisson LM Brizot ML. Risk factors for oxygen requirement in hospitalized pregnant and postpartum women with COVID-19. Clinics (Sao Paulo). (2022) 77:100072. doi: 10.1016/j.clinsp.2022.100072, PMID: 35767901 PMC9212857

[46] De Alencar JCG Sternlicht JM Veiga ADM Marchini JFM Ferreira JC De Carvalho CRR. Timing to intubation COVID-19 patients: can we put it off until tomorrow? Healthcare (Basel). (2022) 10:206. doi: 10.3390/healthcare10020206 35206821 PMC8871804

[47] Gomez UT Francisco RPV Baptista FS Gibelli MABC Ibidi SM Carvalho WB. Impact of SARS-CoV-2 on pregnancy and neonatal outcomes: an open prospective study of pregnant women in Brazil. Clinics (Sao Paulo). (2022) 77:100073. doi: 10.1016/j.clinsp.2022.100073, PMID: 35797767 PMC9234062

[48] Levin AS Freire MP Oliveira MS Nastri ACS Harima LS Perdigao-Neto LV. Correlating drug prescriptions with prognosis in severe COVID-19: first step towards resource management. BMC Med Inform Decis Mak. (2022) 22:246. doi: 10.1186/s12911-022-01983-7, PMID: 36131274 PMC9490728

[49] Marcilio I Lazar Neto F Lazzeri Cortez A Miethke-Morais A Dutilh Novaes HM Possolo de Sousa H. Mortality over time among COVID-19 patients hospitalized during the first surge of the pandemic: a large cohort study. PLoS One. (2022) 17:e0275212. doi: 10.1371/journal.pone.0275212, PMID: 36170328 PMC9518866

[50] Rodrigues DS Nastri ACS Magri MM Oliveira MS Sabino EC Figueiredo PHMF. Predicting the outcome for COVID-19 patients by applying time series classification to electronic health records. BMC Med Inform Decis Mak. (2022) 22:187. doi: 10.1186/s12911-022-01931-5, PMID: 35843930 PMC9288836

[51] Avelino-Silva VI Avelino-Silva TJ Aliberti MJR Ferreira JC Cobello Junior V Silva KR. Prediction of intensive care admission and hospital mortality in COVID-19 patients using demographics and baseline laboratory data. Clinics (Sao Paulo). (2023) 78:100183. doi: 10.1016/j.clinsp.2023.100183, PMID: 36989546 PMC9998300

[52] Taniguchi LU Avelino-Silva TJ Dias MB Jacob-Filho W Aliberti MJR. Group C-aFC-FS, et al. patient-centered outcomes following COVID-19: frailty and disability transitions in critical care survivors. Crit Care Med. (2022) 50:955–63. doi: 10.1097/CCM.0000000000005488, PMID: 35081061 PMC9112506

[53] Silva ECGE Schmitt ACB Godoy CG Gambeta AC Carvalho CRF Fu C. Ambulation capacity, age, immunosuppression, and mechanical ventilation are risk factors of in-hospital death in severe COVID-19: a cohort study. Clinics (Sao Paulo). (2022) 77:100075. doi: 10.1016/j.clinsp.2022.100075, PMID: 35863104 PMC9250925

[54] Astley C Badue Pereira MF Lima MS Buchpiguel CA Carneiro CG Sapienza MT. In-depth cardiovascular and pulmonary assessments in children with multisystem inflammatory syndrome after SARS-CoV-2 infection: a case series study. Physiol Rep. (2022) 10:e15201. doi: 10.14814/phy2.15201, PMID: 35274818 PMC8915155

[55] Battistella LR Imamura M De Pretto LR Van Cauwenbergh SKHAA Delgado Ramos V Saemy Tome Uchiyama S. Long-term functioning status of COVID-19 survivors: a prospective observational evaluation of a cohort of patients surviving hospitalisation. BMJ Open. (2022) 12:e057246. doi: 10.1136/bmjopen-2021-057246, PMID: 35896292 PMC9334693

[56] Busatto GF De Araujo AL Castaldelli-Maia JM Damiano RF Imamura M Guedes BF. Post-acute sequelae of SARS-CoV-2 infection: relationship of central nervous system manifestations with physical disability and systemic inflammation. Psychol Med. (2022) 52:2387–98. doi: 10.1017/S0033291722001374, PMID: 35521752 PMC9151630

[57] Damiano RF Caruso MJG Cincoto AV de Almeida Rocca CC de Padua SA Bacchi P. Post-COVID-19 psychiatric and cognitive morbidity: preliminary findings from a Brazilian cohort study. Gen Hosp Psychiatry. (2022) 75:38–45. doi: 10.1016/j.genhosppsych.2022.01.002, PMID: 35134702 PMC8734055

[58] Damiano RF Neto DB Oliveira JVR Santos JM Alves JVR Guedes BF. Association between chemosensory impairment with neuropsychiatric morbidity in post-acute COVID-19 syndrome: results from a multidisciplinary cohort study. Eur Arch Psychiatry Clin Neurosci. (2022) 273:325–33. doi: 10.1007/s00406-022-01427-3 35633395 PMC9142732

[59] Gil S Gualano B de Araujo AL de Oliveira Junior GN Damiano RF Pinna F. Post-acute sequelae of SARS-CoV-2 associates with physical inactivity in a cohort of COVID-19 survivors. Sci Rep. (2023) 13:215. doi: 10.1038/s41598-022-26888-3, PMID: 36604523 PMC9813883

[60] Imamura M Uchyiama SST Naves GS Abicalaf C Mirisola AR Dos Santos ACA. Ultrasonographic findings in long COVID: a cross-sectional study of 312 patients. Front Med (Lausanne). (2022) 9:1051389. doi: 10.3389/fmed.2022.1051389 36698837 PMC9869060

[61] Freire MP Oliveira MS Magri MMC Tavares BM Marinho I Nastri A. Frequency and factors associated with hospital readmission after COVID-19 hospitalization: the importance of post-COVID diarrhea. Clinics (Sao Paulo). (2022) 77:100061. doi: 10.1016/j.clinsp.2022.100061, PMID: 35728442 PMC9189119

[62] Goncalves NG Aliberti MJR Bertola L Avelino-Silva T Dias MB Apolinario D. Dissipating the fog: cognitive trajectories and risk factors 1 year after COVID-19 hospitalization. Alzheimers Dement. (2023) 19:3771–82. doi: 10.1002/alz.12993 36861807

[63] Leal GN Astley C Lima MS Diniz MFR Lianza AC Sawamura KSS. Segmental cardiac strain assessment by two-dimensional speckle-tracking echocardiography in surviving MIS-c patients: correlations with myocardial flow reserve (MFR) by 13 N-ammonia PET-CT. Microcirculation. (2022) 29:e12750. doi: 10.1111/micc.12750, PMID: 35146846

[64] Astley C Leal GN Gil S Suguita P Fink T Bain V. Home-based exercise training in the recovery of multisystem inflammatory syndrome in children: a case series study. Children (Basel). (2023) 10:889. doi: 10.3390/children10050889 37238437 PMC10217147

[65] Pereira MFB Suguita P Litvinov N Farhat SCL Paula CSY Lazari CDS. SARS-CoV-2 and rhinovirus infections: are there differences in clinical presentation, laboratory abnormalities, and outcomes in the pediatric population? Rev Inst Med Trop Sao Paulo. (2022) 64:e34. doi: 10.1590/s1678-9946202264034 35648987 PMC9134860

[66] Damiano RF Rocca CCA Serafim AP Loftis JM Talib LL Pan PM. Cognitive impairment in long-COVID and its association with persistent dysregulation in inflammatory markers. Front Immunol. (2023) 14:1174020. doi: 10.3389/fimmu.2023.1174020, PMID: 37287969 PMC10242059

[67] Dewulf A Francois G Pahl-Wostl C Taillieu T. A framing approach to cross-disciplinary research collaboration: experiences from a large-scale research project on adaptive water management. Ecol Soc. (2007) 12:214. doi: 10.5751/ES-02142-120214

[68] International Committee of Medical Journal Editors. *Defining the role of authors and contributors*. (2023). Available at: https://www.icmje.org/recommendations/browse/roles-and-responsibilities/defining-the-role-of-authors-and-contributors.html2023.

[69] Provost F Fawcett T. Data science and its relationship to big data and data-driven decision making. Big Data. (2013) 1:51–9. doi: 10.1089/big.2013.1508, PMID: 27447038

[70] Dixon JR Jr. The international conference on harmonization good clinical practice guideline. Qual Assur. (1998) 6:65–74. PMID: 10386329 10.1080/105294199277860

[71] Farber GK Gage S Kemmer D White R. Common measures in mental health: a joint initiative by funders and journals. Lancet Psychiatry. (2023) 10:465–70. doi: 10.1016/S2215-0366(23)00139-6, PMID: 37084745 PMC10198931

[72] Peek N Rodrigues PP. Three controversies in health data science. Int J Data Sci Anal. (2018) 6:261–9. doi: 10.1007/s41060-018-0109-y, PMID: 30957010 PMC6413491

[73] Roussos ST Fawcett SB. A review of collaborative partnerships as a strategy for improving community health. Annu Rev Publ Health. (2000) 21:369–402. doi: 10.1146/annurev.publhealth.21.1.369 10884958

[74] Carfi A Bernabei R Landi F Gemelli Against C-P-ACSG. Persistent symptoms in patients after acute COVID-19. JAMA. (2020) 324:603–5. doi: 10.1001/jama.2020.12603, PMID: 32644129 PMC7349096

[75] Halpin SJ McIvor C Whyatt G Adams A Harvey O McLean L. Postdischarge symptoms and rehabilitation needs in survivors of COVID-19 infection: a cross-sectional evaluation. J Med Virol. (2021) 93:1013–22. doi: 10.1002/jmv.26368, PMID: 32729939

[76] Carvalho-Schneider C Laurent E Lemaignen A Beaufils E Bourbao-Tournois C Laribi S. Follow-up of adults with noncritical COVID-19 two months after symptom onset. Clin Microbiol Infect. (2021) 27:258–63. doi: 10.1016/j.cmi.2020.09.052, PMID: 33031948 PMC7534895

[77] Chopra V Flanders SA O'Malley M Malani AN Prescott HC. Sixty-day outcomes among patients hospitalized with COVID-19. Ann Intern Med. (2021) 174:576–8. doi: 10.7326/M20-5661, PMID: 33175566 PMC7707210

[78] Embi PJ Payne PR. Clinical research informatics: challenges, opportunities and definition for an emerging domain. J Am Med Inform Assoc. (2009) 16:316–27. doi: 10.1197/jamia.M3005, PMID: 19261934 PMC2732242

[79] Richesson RL Krischer J. Data standards in clinical research: gaps, overlaps, challenges and future directions. J Am Med Inform Assoc. (2007) 14:687–96. doi: 10.1197/jamia.M2470, PMID: 17712081 PMC2213488

[80] Chenchula S Vidyasagar K Pathan S Sharma S Chavan MR Bhagavathula AS. Global prevalence and effect of comorbidities and smoking status on severity and mortality of COVID-19 in association with age and gender: a systematic review, meta-analysis and meta-regression. Sci Rep. (2023) 13:6415. doi: 10.1038/s41598-023-33314-9, PMID: 37076543 PMC10115382

[81] Zhang HG Dagliati A Shakeri Hossein Abad Z Xiong X Bonzel CL Xia Z. International electronic health record-derived post-acute sequelae profiles of COVID-19 patients. NPJ Digit Med. (2022) 5:623. doi: 10.1038/s41746-022-00623-8 PMC9242995 35768548

[82] Subramanian A Nirantharakumar K Hughes S Myles P Williams T Gokhale KM. Symptoms and risk factors for long COVID in non-hospitalized adults. Nat Med. (2022) 28:1706–14. doi: 10.1038/s41591-022-01909-w, PMID: 35879616 PMC9388369

[83] Peckham H De Gruijter NM Raine C Radziszewska A Ciurtin C Wedderburn LR. Male sex identified by global COVID-19 meta-analysis as a risk factor for death and ITU admission. Nat Commun. (2020) 11:6317. doi: 10.1038/s41467-020-19741-6 33298944 PMC7726563

[84] Ssentongo P Zhang Y Witmer L Chinchilli VM Ba DM. Association of COVID-19 with diabetes: a systematic review and meta-analysis. Sci Rep. (2022) 12:20191. doi: 10.1038/s41598-022-24185-7, PMID: 36418912 PMC9684130

[85] Tsampasian V Elghazaly H Chattopadhyay R Debski M Naing TKP Garg P. Risk factors associated with post-COVID-19 condition: a systematic review and Meta-analysis. JAMA. Intern Med. (2023) 183:566. doi: 10.1001/jamainternmed.2023.0750 PMC10037203 36951832

[86] O'Mahoney LL Routen A Gillies C Ekezie W Welford A Zhang A. The prevalence and long-term health effects of long Covid among hospitalised and non-hospitalised populations: a systematic review and meta-analysis. EClinicalMedicine. (2023) 55:101762. doi: 10.1016/j.eclinm.2022.101762, PMID: 36474804 PMC9714474

[87] Institute of Medicine. Unequal treatment: Confronting racial and ethnic disparities in health care. Washington, DC: The National Academies Press (2003). 25032386

[88] Chauhan A Walton M Manias E Walpola RL Seale H Latanik M. The safety of health care for ethnic minority patients: a systematic review. Int J Equity Health. (2020) 19:118. doi: 10.1186/s12939-020-01223-2, PMID: 32641040 PMC7346414

[89] Weeks WB Wallace AE Kimberly BC. Changes in authorship patterns in prestigious US medical journals. Soc Sci Med. (2004) 59:1949–54. doi: 10.1016/j.socscimed.2004.02.029, PMID: 15312928

[90] Price WN 2nd Cohen IG. Privacy in the age of medical big data. Nat Med. (2019) 25:37–43. doi: 10.1038/s41591-018-0272-7, PMID: 30617331 PMC6376961

[91] Kohane IS. Using electronic health records to drive discovery in disease genomics. Nat Rev Genet. (2011) 12:417–28. doi: 10.1038/nrg2999, PMID: 21587298

