# Supplementary material for: Data-driven, cross-disciplinary collaboration: lessons learned at the largest academic health center in Latin America during the COVID-19 pandemic
Source: Front Public Health. 2024 Feb 27;12:1369129. doi: 10.3389/fpubh.2024.1369129 (PMC10927964; doi:10.3389/fpubh.2024.1369129)
Supplement: Supplementary file 1 [file Data_Sheet_1.PDF]

Supplementary Methods

Funding allocation

1. Infrastructure

There was a need for investment aimed at improving HC-FMUSP physical infrastructure (cabling and electronic devices) and network to optimize the routine for extracting hospitalization information from electronic health records (EHR). Funds were also allocated for investments in renovations on the building that served as a temporary outpatient center during the follow-up data collection.

2. Third-party service providers

An additional significant allocation of resources involved hiring a team of professional data analytics and data science experts for the organization and integration of data extracted from EHR to construct the institutional databases (including cleaning, structuring, and reconciliation). This team was also responsible to create electronic forms for collecting multidisciplinary follow-up data. The team’s responsibilities also encompassed managing the data, including quality control, ensuring data protection and compliance, and providing necessary technical assistance and support.

3. Equipment and materials for the biobank

Resources were allocated for the acquisition of: (1) an ultra-low temperature freezer to store biobank samples at -80°C, (2) a laboratory centrifuge to process biobank samples, and (3) consumables for collection and processing of these samples.

4. Equipment and materials for follow-up data collection

While the majority of equipment and material resources utilized throughout the follow-up data collection were procured through lending or donation arrangements with the research groups, some specific tests conducted during the multidisciplinary follow-up assessments required investment in individual consumables (e.g., u-Smell-it™ olfactory test).

5. Human resources

Over the course of the initiatives, most actions were voluntarily performed by regularly employed professionals affiliated with HC-FMUSP, including organization and leadership, administrative and office support, data collection, among others. This involved professionals from diverse roles within the institution, including faculty members, other academic staff, students, administrative personnel, and healthcare professionals. However, supplemental resources were essential to uphold the progression of the initiatives.

Resources were allocated to establish a postdoctoral fellowship to assist in the operational management of the research activities, by integrating the hands-on research managing team. The main responsibility of this position was to assist researchers in selecting relevant data fields and facilitate the application of data to their research questions, thereby avoiding redundant analyses, interpretation errors, and inconsistencies in reported data across various papers.

Additionally, while the acquisition of demographic and clinical information during the follow-up assessments was partially managed by voluntary research assistants affiliated with the research groups, complementary funds were needed to allow the hiring of additional healthcare professionals during the peak periods of patient recruitment and assessment.

Lastly, funds were allocated to compensate administrative staff for overtime hours required for subject recruitment and reception during the phase of follow-up assessments and management of the overall logistics for the initiatives.

6. Participant compensation

Funds were utilized to cover for transport expenses and meals for research participants during the follow-up assessments.

7. Costs of publishing papers

Finally, while most of research procedures were voluntarily performed by HC-FMUSP academic personnel and students, resources were assigned to cover publication-related expenses, encompassing fees associated with publishing in open-access journals.

TABLE S1. Cases in our database regarding adult patients (≥ 18 years). Laboratory confirmation of COVID-19 was defined as either: 1) positive reverse-transcriptase polymerase chain reaction (RT-PCR) for SARS-CoV-2 on swab from nasopharyngeal and/or oropharyngeal samples (collected at admission and repeated after 48 hours if negative); or 2) positive testing by chemiluminescent immunoassays to detect serum antibodies, performed for highly suspect cases with at least two negative RT-PCR samples or for whom an RT-PCR test was not available up to day 10 of symptom onset. Patients with nosocomial COVID-19 infections were excluded.

	**Suspected COVID-19 cases**	**Laboratory confirmed COVID-19 cases**	**In-hospital deaths within confirmed COVID-19 cases**	**% of in-hospital deaths within confirmed COVID-19 cases**	**Highly suspected COVID-19 cases with negative diagnostic laboratory results**	**Cases with negative diagnostic laboratory results reviewed as not likely COVID-19 cases**
**March/2020**	66	42	12	28.57%	16	8
**April/2020**	929	640	214	33.44%	190	101
**May/2020**	1143	991	335	33.80%	67	86
**June/2020**	913	783	290	37.04%	41	90
**July/2020**	452	369	134	36.31%	4	79
**August/2020**	250	184	67	36.41%	0	66
**September/2020**	74	45	19	42.22%	2	0
**October/2020**	71	50	14	28.00%	1	0
**November/2020**	82	47	10	21.28%	0	0
**December/2020**	140	93	31	33.33%	0	0
**January/2021**	158	97	26	26.80%	2	0
**February/2021**	131	77	17	22.08%	2	0
**March/2021**	395	255	84	32.94%	3	0
**April/2021**	496	310	94	30.32%	1	0
**May/2021**	394	206	45	21.84%	2	0
**June/2021**	313	160	33	20.63%	1	0
**July/2021**	128	67	11	16.42%	2	0
**August/2021**	81	50	8	16.00%	0	0
**September/2021**	58	30	4	13.33%	0	0
**October/2021**	45	26	7	26.92%	1	0
**November/2021**	25	18	7	38.89%	1	0
**December/2021**	19	5	1	20.00%	0	0
**January/2022**	141	96	25	26.04%	0	0
**February/2022**	43	29	8	27.59%	0	0
**March/2022**	5	3	0	0.00%	0	0
**April/2022**	3	1	1	100.00%	0	0
**May/2022**	12	7	2	28.57%	0	0
**June/2022**	8	5	2	40.00%	0	0
**TOTAL**	**6575**	**4686**	**1501**	**32.03%**	**336**	**430**

TABLE S2. Structure of the hospitalization and multidisciplinary follow-up program databases

Data Collection Form	Aim	Main variables
**Hospitalization database**		
Demographic data	Characterization of the patient's demographic profile	Age<br>Sex<br>Pregnancy information<br>Race<br>Education level<br>Presence of residential exposure to air pollution (calculated from reported address)<br>Residential levels of air pollution and traffic density (calculated from reported address)<br>Residential greenness (calculated from reported address)<br>Healthcare worker (yes/no)
Clinical presentation at hospital admission	Characterization of the patient's health condition at hospital admission	Date of hospital admission<br>Admission via emergency room?<br>Temperature (°C)<br>Heart rate (beats per minute)<br>Respiratory rate (breaths per minute)<br>Systolic blood pressure (mmHg)<br>Diastolic blood pressure (mmHg)<br>Oxygen saturation (%)<br>Fraction of inspired oxygen (FiO2) (%)<br>Simplified Acute Physiology Score (SAPS) 3<br>Weight (kg)<br>Height (cm)<br>General health status at/before hospital admission<br>Comorbidities<br>Medication in use at/before hospital admission<br>Severity of acute illness using the WHO clinical progression scale at hospital admission
Recent symptom history	Characterization of patient's complaint at hospital admission	Date of symptoms onset<br>Time between onset of symptoms and hospital admission (days)<br>Patient's complaint (history of fever, chills, dyspnea/ shortness of breath, cough, cough with sputum production, runny nose, odynophagia, muscular pain/arthralgia, fatigue/tiredness, loss of taste, loss of sense of smell, stroke, headache, alteration in consciousness/confusion, abdominal pain, vomiting, nausea, diarrhea, decreased urination, yellow/dark urine, conjunctivitis, skin rash, other symptoms)
Hospital trajectory	Characterization of the hospital trajectory, complications and hospital outcomes	Length of hospital stay (days)<br>Laboratory confirmation of COVID diagnosis<br>Temperature (°C) after 48 hours of hospitalization<br>Heart rate (beats per minute) after 48 hours of hospitalization<br>Respiratory rate (breaths per minute) after 48 hours of hospitalization<br>Systolic blood pressure after 48 hours of hospitalization<br>Diastolic blood pressure after 48 hours of hospitalization

		Oxygen saturation (%) after 48 hours of hospitalization<br>Temperature (°C) – last measurement during hospitalization<br>Heart rate (beats per minute) – last measurement during hospitalization<br>Respiration rate (breaths per minute) – last measurement during hospitalization<br>Systolic blood pressure – last measurement during hospitalization<br>Diastolic blood pressure – last measurement during hospitalization<br>Oxygen saturation (%) – last measurement during hospitalization<br>Need for vasoactive drugs during hospitalization (yes/no, type, duration)<br>Need for oxygen therapy during hospitalization (yes/no, type, duration)<br>Need for ICU during hospitalization (yes/no, duration)<br>Need for intubation during hospitalization (yes/no, duration)<br>Need for re-intubation during hospitalization (yes/no, duration)<br>Need for prone position (yes/no, duration)<br>Need for replacement renal therapy or dialysis due to acute kidney disease (yes/no, type, duration)<br>Need for ECMO (yes/no, duration)<br>Need for blood products transfusion (yes/no, type)<br>Hospital complications (shock, convulsion, delirium, meningitis/encephalitis, anemia, bleeding, atrial fibrillation, supraventricular arrhythmia, ventricular arrhythmia, cardiac arrest, pneumonia, bronchiolitis, acute respiratory distress syndrome, ischemic stroke, hemorrhagic stroke, digestive hemorrhage, disseminated intravascular coagulation, endocarditis, myocarditis/pericarditis, cardiomyopathy, pancreatitis, liver dysfunction, acute kidney injury, healthcare-related infection, thromboembolic phenomena, other complications)<br>Medications used during hospitalization (description)<br>Hospital outcome (discharge, hospital transfer or death)<br>Date of hospital outcome<br>Mechanism of death (description)
Laboratory test results	Results of laboratory tests done with blood samples collected within first 72 hours of hospitalization and weekly samples	Alanine aminotransferase (ALT)<br>Albumin<br>Amylase<br>Arterial blood gas (ABG)<br>Aspartate aminotransferase (AST)<br>Bicarbonate<br>Brain natriuretic peptide (BNP)<br>C-reactive protein (CRP)<br>Creatine phosphokinase (CPK)<br>Creatinine<br>D-dimer<br>Erythrocyte sedimentation rate (ESR)<br>Ferritin<br>Fibrinogen<br>Free T4 (FT4)<br>Glucose<br>Glutamate pyruvate transaminase (GPT)<br>Hematocrit<br>Hemoglobin<br>Hemoglobin A1C (HbA1c)<br>High-density lipoprotein (HDL)<br>Ionic calcium

		Lactate dehydrogenase (LDH)<br>Lactic acid<br>Leukocytes<br>Lipase<br>Low-density lipoprotein (LDL)<br>Lymphocytes<br>Mean corpuscular hemoglobin (MCH)<br>Magnesium<br>Neutrophils<br>Partial thromboplastin time (PTT)<br>Platelets<br>Potassium<br>Pro-brain natriuretic peptide (pro-BNP)<br>Prothrombin time (PT)<br>Sodium<br>Thyroid-stimulating hormone (TSH)<br>Total calcium<br>Total cholesterol<br>Triglycerides<br>Troponin<br>Urea<br>Uric acid
Laboratory test results – urine	Results of laboratory testing with urine samples collected during hospitalization	Erythrocytes<br>Glucose<br>Ketones<br>Leukocyte esterase<br>Urine protein test
**Multidisciplinary follow-up database**		
Basic information, vital sign and anthropometric measurements	Characterization of the patient’s health condition at the follow-up appointment	Date of follow-up appointment<br>Time (days) from hospital admission to follow-up appointment<br>Time (days) from hospital discharge to follow-up appointment<br>Information on COVID-19 vaccination (yes/no, doses)<br>Temperature (°C) at follow-up appointment<br>Systolic blood pressure (mmHg) at follow-up appointment<br>Diastolic blood pressure (mmHg) at follow-up appointment<br>Resting heart rate (beats per minute) on follow-up appointment<br>Resting respiratory rate (breaths per minute) at follow-up appointment<br>Oxygen saturation (%) at follow-up appointment<br>Weight (kg) at follow-up appointment<br>Height (cm) at follow-up appointment<br>Abdominal circumference (cm) at follow-up appointment<br>Calf circumference (cm) on follow-up appointment<br>Brachial circumference (cm) on follow-up appointment
ELSA-BRAZIL	Baseline semi-structured medical interviewing using the Brazilian longitudinal study of adult health	Sociodemographic characteristics<br>Medical history<br>Occupational history<br>Retirement status (pre-COVID-19 and post-COVID- 19)

		Lifestyle habits (food consumption and smoking)<br>Previous comorbidities (before hospital admission)<br>Current comorbidities (diagnosed after hospital discharge)<br>Current medication use
Current health and patient related symptoms after hospital discharge	Characterization of patient’s complaint at the follow-up appointment	Patient self-rated health status<br>Patient relates SARS-CoV-2 reinfection after hospital discharge<br>Other infections (including respiratory) diagnosed after hospital discharge<br>Emergency room and/or hospital readmission after hospital discharge (date, reason, description)<br>Complaint of persistent COVID-19 symptoms after hospital discharge (yes/no; description)<br>Results of a brief systematic physical and neurological examination
Multidisciplinary evaluation of disability, quality-of-life and overall functioning	Outcome measures from the patient’s perspective (quality of life after hospitalization experience)	Modified Medical Research Council (mMRC) Dyspnea Scale<br>Borg Dyspnea Scale<br>Clinical Frailty Scale<br>Epworth Sleepiness Scale<br>EQ-5D Quality-of-Life Questionnaire – 5-level version (EQ-5D-5L)<br>Functional Assessment of Chronic Illness Therapy (FACIT)<br>Functional Independence Measure<br>Functional Oral Intake Scale (FOIS)<br>Post-COVID-19 Functional Status Scale<br>Insomnia Severity Index<br>International Physical Exercise Questionnaire (short form)<br>Visual-Analogue Scale assessing pain<br>WHO Disability Assessment Schedule 2.0
Structured and/or objective physical tests	Objective measures of patient’s physical functioning at the follow-up appointment	Manual muscle testing using the MRC strength grading system<br>Muscle ultrasound (measurements of muscle thickness (MT) and echo intensity of the anterior rectus muscle and vastus medialis muscle)<br>Measurement of handgrip strength<br>Timed Up and Go test (TUG)<br>1-minute sit-to-stand test<br>10-meters walk test
Olfactory tests	Objective measures of patient’s olfactory functioning at the follow-up appointment	U-Smell it olfactory test<br>Visual-Analogue Scale assessing the impact on quality of life following COVID-related smell and taste loss<br>Visual-Analogue Scale assessing the degree of chemosensitive recovery until the date of the interview
Cognitive test battery	Objective measures of patient’s cognitive functioning at the follow-up appointment	Consortium to Establish a Registry for Alzheimer’s disease (CERAD) battery<br>Digit-symbol test<br>Memory Complaint Scale<br>Mini-Mental State Examination<br>Trail Making Test – part A
Structured instruments to assess mental health status	Structured measures of mental health status at the follow-up appointment	Alcohol Use Disorders Identification Test (AUDIT)<br>Ask Suicide-Screening Questions (ASQ)<br>Clinical Interview Schedule – Revised (CIS-R)<br>Female Sexual Function Index (FSFI)<br>Hospital Anxiety and Depression Scale (HAD)<br>Post-Traumatic Stress Disorder Checklist

		Selected sections from the Structured Clinical Interview for DSM Disorders (SCID)
Pulmonary function tests and chest imaging exams I	Objective measures of patient’s pulmonary functioning at the follow-up appointment (for patients not admitted to the ICU)	Conventional spirometry test<br>Frontal and lateral chest X-ray
Pulmonary function tests and chest imaging exams II	Objective measures of patient’s pulmonary functioning at the follow-up appointment (for patients admitted to the ICU)	Computed tomography scan (CT) imaging of the chest<br>Incremental cardiopulmonary exercise test (CPET)<br>Whole-body plethysmography examination
Laboratory test results	Results of laboratory tests done with blood samples collected at follow-up appointment	Activated partial thromboplastin time (APTT)<br>Alanine aminotransferase (ALT)<br>Albumin<br>Alkaline Phosphatase (ALP)<br>Chloride<br>C-peptide<br>C-reactive protein (CRP)<br>Creatine phosphokinase (CPK)<br>Creatinine<br>D-dimer<br>Ferritin<br>Fibrinogen<br>Free T4 (FT4)<br>Gamma-glutamyl transferase (GGT)<br>Glucose<br>Hematocrit<br>Hemoglobin<br>Hemoglobin A1C (HbA1c)<br>High-density lipoprotein (HDL)<br>Insulin<br>Ionic calcium<br>Iron<br>Lactic acid<br>Lipase<br>Low-density lipoprotein (LDL)<br>Lymphocytes<br>Magnesium<br>Mean corpuscular hemoglobin (MCH)<br>Neutrophils<br>Phosphate<br>Platelets<br>Potassium<br>Pro-brain natriuretic peptide (pro-BNP)<br>Prothrombin time (PT)<br>Sodium<br>Thyroid-stimulating hormone (TSH)<br>Total calcium<br>Total cholesterol<br>Total iron-binding capacity (TIBC)<br>Total protein<br>Transferrin saturation (TS)<br>Triglycerides

		Troponin<br>Urea
Laboratory test results – urine	Results of laboratory testing with urine samples collected at follow-up appointment	Crystals<br>Erythrocytes<br>Glucose<br>Ketones<br>Leukocyte esterase<br>Nitrites<br>Urine creatinine<br>Urine density<br>Urine pH<br>Urine protein test

Abbreviations: °C: degree Celsius; mmHg: millimeter of mercury; kg: kilograms; cm: centimeters; ICU: intensive care unit; ECMO: extracorporeal membrane oxygenation; WHO: World Health Organization; MRC: Medical Research Council.

TABLE S3. Research investigations currently using the HC-FMUSP Institutional Database of hospitalizations due to COVID-19

Research topic	Funding agencies	Internal leadership	External groups	Status
Protective ventilation and outcomes of critically ill patients with COVID-19	----	Pulmonary & Critical Care	----	Published (Ferreira et al., Ann Intensive Care 2021)
COVID-19-related hospital cost-outcome analysis	----	Clinical Director's Office	----	Published (Miethke-Morais et al., *Braz J Infect Dis* 2021)
Differences in children and adolescents with SARS-CoV-2 infection	CNPq, FAPESP	Pediatrics	----	Published (Marques et al., *Clinics* 2021)
Use and misuse of biomarkers and the role of D-dimer and C-reactive protein in the management of COVID-19	----	Laboratory & Pathology	----	Published (Gonçalves et al., *Clinics* 2021)
Using frailty for prognostication in hospitalized patients with COVID-19	----	Geriatrics	----	Published (Aliberti et al., *J Am Geriatr Soc* 2021)
Vertical transmission of SARS-CoV2 during pregnancy	CAPES, Horizon 2020 (EU)	Obstetrics	Center for Global Health, Colorado School of Public Health, USA	Published (Maeda et al., *Prenat Diagn* 2021)
Muscle strength and muscle mass as predictors of hospital length of stay in patients with moderate to severe COVID-19	CAPES, CNPq, FAPESP	Rheumatology	EEFE-USP, Brazil; School of Health Sciences, Aberdeen, UK	Published (Gil et al., *J Cachexia Sarcopenia Muscle* 2021)
Timing to intubation in COVID-19 patients	FAPESP	Emergency care	IME-USP, Brazil	Published (de Alencar et al., *Healthcare* 2022)
Distinct outcomes in COVID-19 patients with positive or negative RT-PCR test	FAPESP	Emergency care	IME-USP, Brazil	Published (Menezes et al., *Viruses* 2022)
Patient-centered outcomes following COVID-19	----	Geriatrics	----	Published (Taniguchi et al., *Crit Care Med* 2022)
Risk factors for oxygen requirement in hospitalized pregnant and postpartum women with COVID-19	CAPES	Obstetrics	----	Published (Baptista et al., *Clinics* 2022)
Impact of COVID-19 on pregnancy and neonates	CAPES, Horizon 2020 (EU)	Obstetrics	Center for Global Health, Colorado School of Public Health, USA	Published (Gomez et al., *Clinics* 2022)
Predicting the outcome for COVID-19 patients by applying time series classification to electronic health records	FAPESP	Infectious diseases	Laboratory of Computer Applications for Health Care, USP, Brazil	Published (Rodrigues et al., *BMC Med Inform Decis Mak* 2022)
Mortality over time among COVID-19 patients hospitalized during the first surge of the pandemic	----	Epidemiology		Published (Marcilio et al., *PLoS One* 2022)
Correlating drug prescriptions with prognosis in severe COVID-19	FAPESP	Infectious diseases	Laboratory of Computer Applications for Health Care, USP, Brazil	Published (Levin et al., *BMC Med Inform Decis Mak* 2022)
Ambulation capacity, age, immunosuppression, and mechanical ventilation are risk factors of in-hospital death in severe COVID-19: a cohort study	----	Pulmonary & Physical Therapy	----	Published (Silva et al., *Clinics* 2022)
Prediction of intensive care admission and hospital mortality in COVID-19 patients using demographics and baseline laboratory data	----	Infectious diseases	----	Published (Avelino-Silva et al., *Clinics* 2023)
Assessment of troponin and cardiovascular comorbidities as prognostic markers in patients hospitalized for COVID-19	----	Cardiology	----	Manuscript under preparation
Retrospective cohort study comparing the first and second COVID-19 waves	FAPESP	Emergency care	----	Manuscript under preparation
Prognosis of COVID-19 elderly patients to inform discussions of values-based care	FAPESP	Emergency care	----	Manuscript under preparation
Diabetes related phenotypes and their influence on outcomes of patients with COVID-19	----	Endocrinology	Department of Genetics & Evolutionary Biology, USP, Brazil	Manuscript under preparation

Abbreviations: BMGF: Bill & Melinda Gates Foundation; IME: *Instituto de Matemática e Estatística* (Institute of Mathematics and Statistics); CAPES: *Coordenação de Aperfeiçoamento de Pessoal de Nível Superior* (Brazilian Coordination for the Improvement of Higher Education Personnel); CNPq: *Conselho Nacional de Desenvolvimento Científico e Tecnológico* (Brazilian National Council for Scientific and Technological Development); EEFE: *Escola de Educação Física e Esporte* (School of Physical Education and Sports); EU: European Union; FAPESP: *Fundação de Amparo à Pesquisa do Estado de São Paulo* (São Paulo Research Foundation); USP: Universidade de São Paulo.

TABLE S4. Research investigations currently using the HC-FMUSP Institutional Database of multidisciplinary follow-up assessments after in-hospital discharge

Research topic	Funding agencies	Internal groups involved	External groups	Status
Persistent symptoms and decreased health-related quality of life after symptomatic pediatric COVID-19	CNPq, FAPESP	Pediatrics, Rheumatology, Radiology, Pulmonary & Critical Care, Physical Medicine & Rehabilitation, Cancer Metabolism Research Group, Psychiatry	----	Published (Fink et al., *Clinics* 2021)
Post-COVID-19 psychiatric and cognitive morbidity	----	Psychiatry, Neurology	Massachusetts General Hospital and Harvard Medical School, USA	Published (Damiano, Caruso, et al., *Gen Hosp Psychiatry* 2022)
Clinical, sociodemographic and environmental factors impact post-COVID-19 syndrome	----	Epidemiology, Pathology, Pulmonary & Critical Care, Infectious Diseases, Emergency care, Physical Medicine & Rehabilitation, Psychiatry, Neurology, Internal Medicine, Radiology, Otorhinolaryngology	----	Published (Ferreira et al., *J Glob Health* 2022)
Long-term functioning status of COVID-19 survivors	FAPESP	Physical Medicine & Rehabilitation	Massachusetts General Hospital and Harvard Medical School, USA	Published (Battistella et al., *BMJ Open* 2022)
Association between chemosensory impairment with neuropsychiatric morbidity in post-acute COVID-19 syndrome	----	Otorhinolaryngology, Psychiatry, Neurology	----	Published (Damiano, Neto, et al., *Eur Arch Psychiatry Clin Neurosci* 2022)
Predictive clinical model for chronic lung lesions in COVID-19 survivors	FAPESP	Pulmonary & Critical Care, Radiology, Information Technology		Published (Carvalho et al., *BMJ Open* 2022)
Relationship of central nervous system manifestations with physical disability and systemic inflammation in long COVID	----	Psychiatry, Pulmonary & Critical Care, Infectious Diseases, Emergency care, Physical Medicine & Rehabilitation, Neurology, Internal Medicine, Radiology, Otorhinolaryngology	----	Published (Busatto et al., *Psychol Med* 2022)
Frequency and factors associated with hospital readmission after COVID-19 hospitalization	----	Infectious Diseases, Epidemiology, Psychiatry	----	Published (Freire et al., *Clinics* 2022)
Cardiovascular and pulmonary evaluations in children with multisystemic inflammatory syndrome after SARS-CoV-2 infection	CNPq, FAPESP	Pediatrics, Rheumatology, Radiology, Pulmonary & Critical Care	----	Published (Astley et al., *Physiol Rep* 2022)
Segmental cardiac strain assessment by two-dimensional speckle-tracking echocardiography in surviving multisystem inflammatory syndrome in children	CNPq, FAPESP	Pediatrics, Rheumatology, Radiology, Oncology and School of Physical Education and Sport	----	Published (Leal et al., *Microcirculation 2022*)
Ultrasonographic evaluation in long COVID	----	Physical Medicine & Rehabilitation	----	Published (Imamura et al., *Front Med* 2022)
Post-acute sequelae of SARS-CoV-2 associates with physical inactivity in a cohort of COVID-19 survivors	CNPq, FAPESP	Rheumatology, Pulmonary & Critical Care, Physical Medicine & Rehabilitation, Psychiatry	----	Published (Gil et al., *Sci Rep* 2023)
Long-term respiratory follow-up of ICU hospitalized COVID-19 patients	FAPESP	Pulmonary & Critical Care, Radiology, Information Technology	----	Published (Carvalho et al., *PLoS One* 2023)
Dissipating the fog: Cognitive trajectories and risk factors 1 year after COVID-19 hospitalization	----	Geriatrics, Pathology, Psychiatry, Neurology, Internal Medicine	----	(Gonçalves et al., *Alzheimers Dement* 2023)
Home-based exercise training in the recovery of multisystem inflammatory syndrome in children: a case series study	CNPq, FAPESP	Pediatrics, Rheumatology, Radiology, Oncology and School of Physical Education and Sport	----	Published (Astley et al., *Children (Basel)* 2023)

Abbreviations: CNPq: *Conselho Nacional de Desenvolvimento Científico e Tecnológico* (Brazilian National Council for Scientific and Technological Development); FAPESP: *Fundação de Amparo à Pesquisa do Estado de São Paulo* (São Paulo Research Foundation).

TABLE S5. Research projects currently using resources from the HC-FMUSP COVID-19 Biobank

Total number of proposed studies	21
Total number of blood aliquots dispensed	approximately 3700
Total number of aliquots planned for approved studies	approximately 4100
Percentage of studies based solely on blood samples collected during hospitalization	42.9%
Percentage of studies based solely on blood samples collected during follow-up assessments	47.6%
Percentage of studies based on blood samples collected during both hospitalization and follow-up	9.5%
Internal groups involved	N = 16 (Cancer Metabolism Research Group, Cardiology, Emergency care, Immunology, Infectious diseases, Internal Medicine, Laboratory & Pathology, Molecular Biology, Neurology, Pathology, Pediatrics, Physical Medicine & Rehabilitation, Psychiatry, Pulmonary & Critical Care, Radiology, Virology)
Total number of external groups involved	11
Published papers	2 (Pereira et al., *Rev Inst Med Trop Sao Paulo* 2022; Damiano et al., *Front. Immunol*. 2023)
Manuscripts submitted for publications	2
